# Evaluation of end-to-end 3D absorbed dose distribution in ^90^Y-SIRT and SBRT combination therapy using MAGIC-f polymer gel dosimeter

**DOI:** 10.1007/s00259-025-07461-2

**Published:** 2025-08-08

**Authors:** Zahra Mansouri, Yazdan Salimi, Nikolaos Koutsouvelis, Habib Zaidi

**Affiliations:** 1 Division of Nuclear Medicine and Molecular Imaging, Diagnostic Department, Geneva University Hospital, Geneva, Switzerland; 2 Division of Radiation Oncology, Geneva University Hospital, Geneva, Switzerland; 3 Department of Nuclear Medicine and Molecular Imaging, University of Groningen, University Medical Center Groningen, Groningen, Netherlands; 4 Department of Nuclear Medicine, University of Southern Denmark, Odense, Denmark; 5 University Research and Innovation Center, Óbuda University, Budapest, Hungary

**Keywords:** ^90^Y-SIRT, SBRT, Gel dosimetry, MAGIC-f, Combination therapy, 3D dosimetry

## Abstract

**Background:**

Selective Internal Radiation Therapy (SIRT) with ^90^Y-microspheres, and Stereotactic Body Radiation Therapy (SBRT), are well-established treatment strategies for liver malignancies. Combining these two modalities has proven to be an effective and safe approach for addressing undertreated tumor regions from initial SIRT through a boost dose from SBRT. The complex dosimetry process, which includes image-based dosimetry of ^90^Y-SIRT and SBRT dose painting, requires precise dosimetry verification. In this study, gel dosimetry is proposed as a valuable novel tool to capture the 3D absorbed dose contributions from both treatments with high spatial resolution.

**Methods:**

The MAGIC-f polymer gel dosimeter was used in three experiments: external beam radiation therapy (EBRT), ^90^Y-SIRT, and combination therapy. For EBRT, eight calibration vials filled with gel were irradiated with absorbed doses ranging from 0 to 21 Gy. In the ^90^Y-SIRT experiments, eleven vials were filled with gel containing varying concentrations of ^90^Y-citrate, delivering doses from 0 to 44 Gy after 68 h. For combination therapy, eight vials received 0–7 Gy from ^90^Y-citrate after 68 h, followed by an additional 0–7 Gy from SBRT (final doses 0–14 Gy). A phantom, designed to simulate a tumor within a normal liver, was created with the sphere receiving 10 Gy after 68 h from SIRT and another 10 Gy from SBRT using 6MV photons (in total 20 Gy). PET/CT imaging was conducted before SBRT, and the Medical Internal Radiation Dose (MIRD) schema was used for dose calculations. For each experiment, MR T2-weighted imaging was performed using a 3 Tesla scanner, and R2 maps (1/s) were analyzed to establish a dose-response relationship between absorbed dose (Gy) and R2. The gel’s sensitivity to each irradiation was measured, and phantom’s dose maps were evaluated using mean absorbed dose, dose volume histograms (DVHs), line profiles, and isodose maps.

**Results:**

The dose response of the gel was linear within the irradiated ranges for EBRT and combination therapy. The linear range for ^90^Y-SIRT was between 0 and 16.75 Gy. The sensitivity of the gel was 0.380, 0.758, and 0.713 s⁻¹/Gy for EBRT, ^90^Y-SIRT, and combination experiments, respectively. In the phantom irradiated with combination therapy, a saturated area was observed in the central core of the sphere, surrounded by an underestimation area with a diameter of 4.5 mm. The mean absorbed dose values within the sphere were 9.83 Gy, 9.71 Gy, and 18.58 Gy from SBRT, SIRT, and combination therapy, respectively. For the cylinder, these values were 1.29 Gy, 0.61 Gy, and 2.68 Gy, respectively. The DVHs, line profiles, and isodose lines for the combination therapy demonstrated the cumulative effects of the absorbed dose from both treatments.

**Conclusion:**

This is the first study demonstrating the feasibility of using MAGIC-f gel dosimetry to directly measure 3D dose distributions from combined ^90^Y-SIRT and SBRT. Compared to PET/CT-based dosimetry, the gel method offers superior spatial resolution and enables objective physical verification of complex dose distributions. This technique has strong potential for quality assurance in theranostic protocols, particularly for beta emitters and, potentially, alpha-emitting radiotracers in future applications.

**Supplementary Information:**

The online version contains supplementary material available at 10.1007/s00259-025-07461-2.

## Introduction

Stereotactic body radiation therapy (SBRT) and selective internal radiation therapy (SIRT) are advanced, highly targeted treatment modalities for primary and secondary hepatic malignancies [1, 2]. SBRT is an advanced form of external beam radiotherapy (EBRT), that delivers hypo-fractionated, highly conformal, and high dose radiation to the target volumes. In contrast, SIRT is an internal radiotherapy technique, involving the injection of ^90^Y-microspheres directly into the tumors via its arterial blood supply.

Combining SBRT and SIRT can enhance their therapeutic efficacy and minimize the adverse effects by protecting normal tissues [3]. Current indications are in scenarios where tumor sizes or location limits the safe administration of SBRT alone without exceeding dose constraints for surrounding healthy tissue, therefore, SIRT is employed first to debulk the tumor, followed by a boost from SBRT to target any residual disease [4–6].

Despite the promise of combination therapy, it is not currently supported by consensus guidelines. A significant barrier to this combination is the absence of a standardized dosimetry framework [7, 8]. While dosimetry calculations for combination therapy have utilized biologically effective dose (BED) metrics, the parameters necessary for ^90^Y-BED calculations are often extrapolated from EBRT which are not suitable for SIRT dosimetry [5, 9]. Recent advances in dosimetry techniques and tumor control probability (TCP) modeling have led to the development of BED models and parameters tailored for SIRT [10]. However, these models are primarily based on retrospective studies with limited sample sizes and require further validation for broader clinical applicability. Efforts have also been made to establish approaches for direct use of ^90^Y PET/CT dose maps following SIRT for SBRT boost planning [4, 6]. In these approaches, post-SIRT dosimetry relies on either bremsstrahlung SPECT/CT or PET/CT to generate 3D voxel-based absorbed dose maps and calculate mean absorbed doses to individual lesions.

SPECT or PET image-based dosimetry involves a series of distinct procedures, including the calibration of PET or SPECT scanners, activity measurements, quantitative imaging, and the conversion of activity concentration to absorbed dose values. Each of these steps introduces its own uncertainties, and currently, there is no standardized methodology across the entire process. Moreover, the resolution of dose maps calculated from PET and SPECT is lower than dose resolution of SBRT [11], leading to further inaccuracies when integrating dose maps from these imaging modalities with those generated by SBRT.

Currently, no dedicated tool exists for internal dosimetry to provide direct and tangible absorbed dose measurements, as current dosimetry relies solely on image-based methods. On the other hand, conventional and routine dosimetry tools available in EBRT are inadequate for this purpose as they are neither true three-dimensional (3D) tools nor are they able to measure cumulative doses over multiple exposures of different irradiation types and qualities.

A dosimetry tool that meets all the aforementioned criteria and is well-suited for this purpose is the 3D gel dosimeter, which undergoes structural changes proportional to the absorbed dose when exposed to ionizing radiation [12–14]. Among the various gel dosimeters, polymer gel dosimeters (PGDs) show great promise for the future of dosimetry [12].

PGDs are well-regarded for their stable dose-response, high spatial resolution, independency from radiation orientation, dose rate, and quality, soft tissue equivalency, ease of production and handling, and their compatibility with a wide range of imaging systems, including magnetic resonance imaging (MRI), computed tomography (CT), ultrasound, Raman spectroscopy and optical CT for their read-out [12, 15]. The capabilities of PGDs have been evaluated in various modalities of brachytherapy [16–18] and EBRT [19–25], including SBRT [22, 26]. However, only a limited number of studies have explored their potential applications in internal dosimetry for ^99m^Tc, ^32^P, ^131^I [27–32]. The potential of gel dosimetry in theranostics warrants further evaluation, particularly for newly developed radionuclides and emerging treatments, such as combination therapies involving two different types of irradiations. To the best of our knowledge, there is a lack of comprehensive studies investigating specifically the use of PGDs in SIRT or combination therapy contexts. This study investigated the role of a PGD, MAGIC-f [33, 34], in recording the dose distributions from SIRT alone, SBRT alone, and assess its suitability for complex dosimetry scenarios involving multimodal treatments, such as SIRT + SBRT combination therapy.

## Materials and methods

**Fig. 1:**
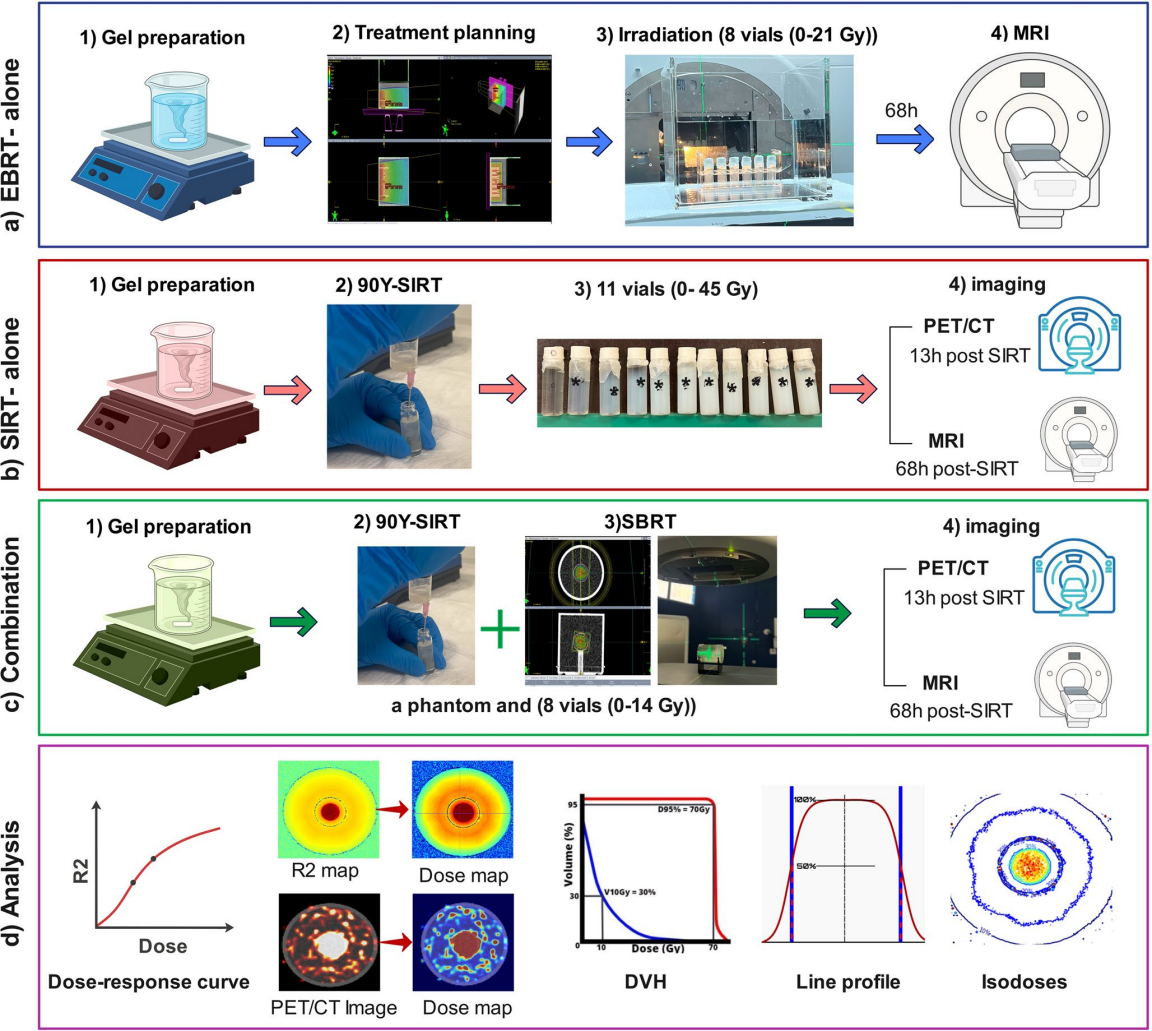
This study comprised 3 different parts. (**a**) The potential of MAGIC-f polymer gel dosimeter in “EBRT-alone”. After preparing the gel, vials were irradiated using EBRT 6MV photon homogeneously. Gel read-out was performed using 3 T MRI. (**b**) “SIRT-alone”; vials were filled with a thoroughly mixed gel with different amounts of ^90^Y activity concentrations to create different absorbed doses (Gy). The gel samples then underwent PET/CT imaging and read-out by MRI. (**c**) “Combination”; The calibration vials and a phantom were filled with thoroughly mixed gel with different amounts of ^90^Y activities. Then, the phantom was irradiated with SBRT technique using 6MV flattening-filter-free photons, and calibration vials irradiated using a homogenous 6MV beam. The gel samples then underwent PET/CT imaging and read-out by MRI. (**d**) analysis: R2 mapping was conducted and SIRT dosimetry using the MIRD schema was applied on PET/CT images. The dose maps were evaluated using different evaluation metrics

Figure 1 illustrates the flowchart summarizing the entire study.

### Phantom design

A phantom was constructed to simulate the liver and a detectable lesion for dose measurement and imaging studies. The design and material are compatible with various imaging modalities and irradiation procedures. All compartments are made of transparent polymethyl methacrylate (PMMA), having a density similar to water (~ 1.1 g/cm³).

The primary phantom comprises a cylindrical compartment representing the whole liver, with an external height of 11 cm and external diameter of 9 cm. This cylinder encompasses a sphere component simulating a lesion. The wall-thickness of the cylinder is 5 mm (internal diameter = 8 cm, internal height = 10 cm). The total volume of the cylinder, in presence of all compartments, is 453.5 ml.

Within the cylinder, the spherical component representing a lesion has an internal diameter of 2.7 cm, allowing it to be detectable even with a low-spatial resolution scanner, such as SPECT. The distance from the rim of the sphere to the lid of cylinder is 6.2 cm, and the volume of the sphere is 10.5 ml.

**Fig. 2:**
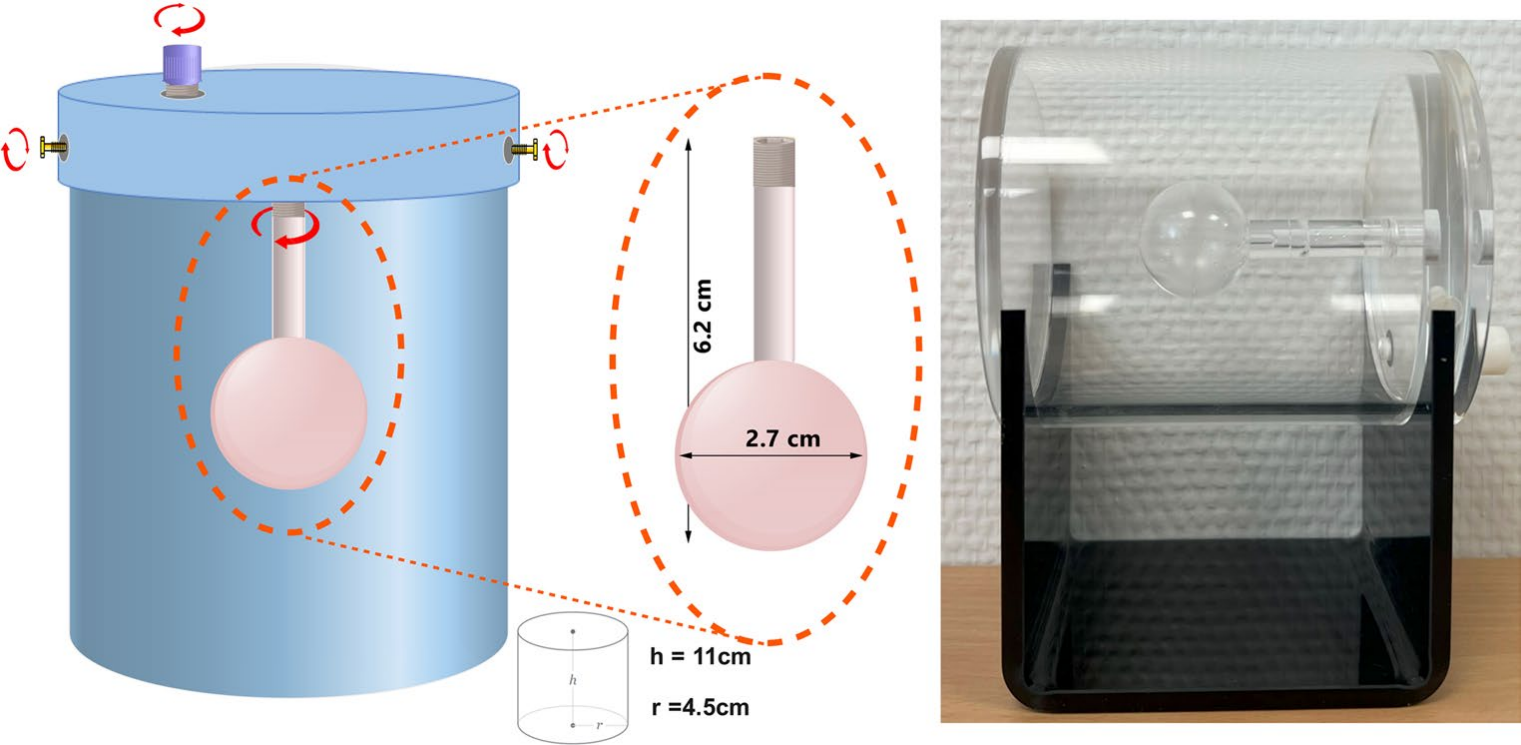
Left: Schematic representation of the phantom; Right: The actual phantom on its holder

The lid of the cylinder can be detached using two plastic screws, and an additional screw on the top facilitates the filling of the phantom. The sphere and its shaft are detachable to allow easier handling. Moreover, the lid has an internal edge extent that fits inside the cylinder to prevent leakage. The lid thickness is 1 cm. A holder is designed for the phantom to assist with immobilization during EBRT irradiation and storing the gel. The height from the center of the gel to the base of the holder is 10 cm. Figure 2 presents a schematic view and the actual phantom with specified dimensions

### MAGIC-f polymer gel preparation

Methacrylic Ascorbic acid gel initiated by copper with formaldehyde (MAGIC-f) PGD was selected for this study for four main reasons: (i) it is a normoxic gel, meaning it can be produced in the presence of atmospheric oxygen, and (ii) its high melting point of 69 °C allows for convenient handling and storage; (iii) its dose-response is stable up to 90 days, and (iv) very small dependency to the dose-rate [33].

When exposed to ionizing radiation, free radicals (e.g., hydroxyl radicals, hydrogen atoms) are formed within the gel dosimeters via the radiolysis of water. These radicals initiate polymerization of the monomers present in the gel. The extent of polymerization is proportional to the local absorbed radiation dose. MAGIC-f consists of methacrylic acid as monomers that undergo radiation-induced polymerization proportional to absorbed dose. Gelatin provides the gel matrix for polymerization, keeping the monomers in place. Formaldehyde is a cross-linker between the polymerized chains. Copper sulfate serves as a catalyst or initiator for radical formation. MAGIC-f was prepared in different amounts for each experiment described in the following sections, according to the method described by Pavoni et al. [33]. The chemical composition of MAGIC-f gel and their mass percentages used in this study is reported in Table 1. Gelatin was added to water at room temperature, and after soaking, it was heated on a hotplate to 45 °C. The mixture was stirred using a magnetic stirrer for approximately 30 min until the gelatin was fully dissolved. The heat was turned off, but stirring continued until the solution cooled to 35 °C. At this point, ascorbic acid, copper sulfate, and formaldehyde were added. After 5 min of mixing, methacrylic acid was introduced, and once thoroughly mixed for 5 min, the polymer gel was ready.

**Table 1:** MAGIC-f gel components and their mass concentration

Component	Mass concentration (%)
Mili-Q Water (Thermo Fisher Scientific^®^)	82.31
Porcine skin gelatin (Type A)– 300 Bloom (Sigma Aldrich^®^)	8.33
Methacrylic acid stabilized with hydroquinone monomethyl ether (Sigma Aldrich^®^)	5.99
formaldehyde, a water solution with 37% minimum and stabilized with 10% methanol (Sigma Aldrich^®^)	3.32
Ascorbic acid (Thermo Fisher Scientific^®^)	0.03
Copper sulfate (Merck^®^)	0.02

After pouring the gel into the containers, the gel samples were stored in a refrigerator at 4 °C.

### EBRT_alone_ irradiation

Before experiments involving EBRT or SBRT, the water-filled vials and phantom underwent planning CT, as detailed in the Supplemental section. For this part of the study, around 100 ml gel was prepared to fill eight calibration vials. planning CT images were imported into Varian Eclipse™ Treatment Planning System (TPS) version 16.1 (Varian Medical Systems, Palo Alto, CA) to calculate the monitor units for irradiation. The calibration vials were arranged in a rack and were immersed in the water tank similar to planning CT setup. A Varian TrueBeam linear accelerator was used for irradiation. An irradiation field of 20 × 10 cm² was set to ensure adequate coverage of the vials. The center of the vials was at a depth of 5 cm (source-to-skin distance, SSD = 95) cm to ensure homogenous irradiation. A 6 MV photon beam with a dose rate of 600 MU/min was used to irradiate the vials from 0 to 21 Gy, with increments of 3 Gy. The gantry angle was set to 270. The vials were irradiated at 600 MU/min to match the average dose rate used in the SBRT treatment plan for the combination study. After irradiation, they were refrigerated until MR imaging was performed.

### SIRT_alone_ irradiation

A total of 300 mL of gel was prepared for this experiment, with 2% reduction in water content to account for the diluted injected activity (further elaborated in the supplementary section). Eleven absorbed dose levels (0, 1.86, 3.72, 5.58, 11.16, 16.75, 22.33, 27.91, 33.5, 39.08, and 44.66 Gy) were considered to prepare the calibration vials. A wide calibration range up to ~ 45 Gy was used to identify linear dynamic range in response to ^90^Y. The absorbed dose was calculated using the Medical Internal Radiation Dose (MIRD) schema (Eq. 1):

1 $$\:D\left(target\right)=\frac{DCF\times\:{A}_{0}\:\left(Target\right)\left[GBq\right]}{M\left(Target\right)\left[Kg\right]}$$

where dose conversion factor (DCF) is 49.77 J/GBq (MIRD Pamphlet No. 29) [35], A_0_(Target) is the required activity (GBq), and M (target) is the target mass (kg).

DCF = 49.77 J/GBq is defined based on the assumption of decay to infinity by integrating the total energy emitted by all beta decays of ^90^Y over its entire decay process. Since our objective was to measure the gel dosimeters 68 h post-irradiation, we adjusted the activity calculations by integrating a ratio of the area under the curve (AUC-ratio, Eq. 2) from the injection time (t = 0, the time we added the radiotracer to the gel) to t = 68 h.

2 $$\:AUC-ratio=(1-{e}^{-\lambda\:{T}_{img}})$$

where:

AUC-ratio: (Area under the curve till time T_img_/area under the curve till infinite time)

𝜆: The decay constant (𝜆=ln (2)/𝑇_1/2,_ where 𝑇_1/2_ is the half-life of ^90^Y = 64.1 h).

T_img_: imaging time as the time from calibrated activities to MR imaging; in this study, T_img_=68 h.

The calibration vials underwent PET/CT imaging using a Siemens Biograph Vision.X (Siemens Healthineers, Erlangen, Germany) at Geneva University Hospital [36], 13 h post-injection. The details of dilution, filling the vials, and imaging are provided in supplementary section.

### Combination therapy

A total of 850 ml of gel (with 2% reduced water content) was prepared to fill eight calibration vials and the phantom. Following the MIRD schema, as in the SIRT_alone_ part, the target was to create 8 calibration vials with absorbed dose values ranging from 0 to 7 Gy (in 1 Gy increments) from SIRT after 68 h, and then an additional 0–7 Gy from SBRT. This resulted in eight vials with final absorbed doses of 0, 2, 4, 6, 8, 10, 12, and 14 Gy with equal contributions from SIRT and SBRT. The SBRT irradiation (0–7 Gy) was performed as described in “EBRT_alone_ irradiation” section.

The phantom was filled in a way so that the sphere received an absorbed dose of 9.999 Gy (activity concentration of 0.388 MBq/mL measured by the dose calibrator), and the cylinder received 0.637 Gy (activity concentration of 0.019 MBq/mL measured by the dose calibrator) both after 69 h. The samples were shielded and stored in a refrigerator for 22 h before undergoing SBRT irradiation. This time was required for gelation and to observe gel’s color-change due to SIRT irradiation.

SBRT irradiation of the phantom involved performing inverse planning for photon using Varian Eclipse™ TPS, version 16.1 (Varian Medical Systems, Palo Alto, CA). The planning target volume (PTV) was manually delineated within the spherical region. The dose distribution for the treatment plan was calculated and optimized using Photon Optimizer version 16.1 and the Anisotropic Analytical Algorithm (AAA) version 15.6, with a grid size of 1 mm for high calculation resolution.

The gel phantom was irradiated in a single-fraction setup with 6 MV flattening-filter-free (FFF) coplanar dual full arcs, delivered using a Varian TrueBeam linear accelerator with a standard Millennium 120 leaf multi-leaf collimator. A prescribed dose of 10 Gy was delivered to the PTV (PTV mean dose), using VMAT technique at a variable dose rate up to 1400 MU/min (on average 600 MU/min). The calculated dose distribution from the TPS was exported as RTDose DICOM files for subsequent analysis. For more details see supplementary material and Figure S1.

### Gel read out: MR imaging

MR imaging of the phantom and corresponding vials was performed 68 h post-irradiation after each experiment. This time interval was necessary to ensure the completion of the polymerization process and to maintain consistency across experiments. For the SIRT and combination therapy phantoms, this delay also allowed sufficient decay of the radionuclide. Imaging was conducted on a Siemens Magnetom Prisma 3 T MRI scanner (Siemens Healthineers, Erlangen, Germany). To ensure temperature stability, the samples were placed inside the MRI room for 2 h prior to imaging. The samples were positioned at the center of a 64-channel head coil, which functioned as both the transmitter and receiver. The vials, secured in a plexiglass vial holder, and the phantom was secured on its plastic holder. The samples with their holders were immersed in a water-filled plastic container to minimize susceptibility artifacts and for consistency in temperature. A multi-spin echo sequence was employed to acquire T2-weighted images. The imaging parameters are reported in Table 2. DICOM files of the MR images were exported for further analysis.

**Table 2:** MR imaging parameters used in this study. *Distance factor = 0 means that there was no gap between the MR slices

Specification	Value
Field of view (FOV)	111 × 111 mm^2^
Time to first echo (TE_1_)	22.5 ms
Repetition time (TR)	3000 ms
Slice thickness	2 mm
Matrix size	256 × 256 voxels
Echo spacing	22.5 ms
Echo train length	360 ms
Number of echoes	16
Number of excitations (NEX)	2
Distance factor *	0

### Post-imaging analysis

PET/CT images from both SIRT_only_ or combination treatments were used to validate dosimetry in the vials and phantom. Activity measurements were corrected for the time delay between SIRT and MRI acquisition. Voxel-level dosimetry was performed on the phantom using the MIRD schema, converting PET-derived activity concentration at the time of gel filling (in Bq/ml) into absorbed dose maps. This calculation accounted for the ^90^Y half-life (64.1 h), gel density (1.03 g/cm³), and using the activity concentration within the entire phantom for self-calibration. The methodology is detailed in our previous works [37–39].

MR images from each experiment were analyzed. R2 maps (1/T2) were generated from images acquired at different echo times (TE) using an in-house Python code (available on the lab’s GitHub). A mono-exponential relationship between MR signal and TE was established using 16 echoes. However, the first echo time was discarded due to its susceptibility to errors caused by B1 inhomogeneities and imperfect refocusing pulses [40]. The remaining 15 echoes were then used to fit the mono-exponential curve.

The average of R2 values within each vial and the corresponding dose value were used to generate dose-response curves for the polymer gel. PET and TPS-derived dose maps were rigidly registered to those calculated from MRI (used as reference) manually using ITK-SNAP 4.0, guided by the CT portion of the PET/CT and the CT simulation from SBRT. The sphere (tumor) and cylinder (normal liver, excluding sphere) regions were manually segmented on MRI, and visually checked on the CT portion of the PET/CT or planning CT of the TPS to ensure proper coverage. The mean absorbed doses from each dose map was calculated for each segmentation. Moreover, we derived the dose volume histograms, line profiles, and isodose of each dose map within the sphere and cylinder.

We also quantified dose inhomogeneity resulting from the non-uniform or colloidal distribution of ^90^Y (calculated from PET) and compared it with the inhomogeneity observed in SBRT (calculated by TPS). The metrics used included the coefficient of variation (CV) and the Heterogeneity Index (HI) of dose values within the tumor (sphere) and the normal liver tissue (cylinder excluding the sphere) VOIs, as defined by the following equations:

3 $$\:\mathrm{C}\mathrm{V}=\raisebox{1ex}{${\upsigma\:}$}\!\left/\:\!\raisebox{-1ex}{${\upmu\:}$}\right.$$

where $\:{\upsigma\:}$ denotes the standard deviation of the dose values within the VOI, and $\:{\upmu\:}$ represents the mean absorbed dose within the VOI.

4 $$\:\mathrm{H}\mathrm{I}=\frac{\mathrm{D}_{2\%}-\mathrm{D}_{98\%}}{\mathrm{D}_{50\%}}$$

where D_x%_ (Gy) denotes the absorbed dose received by x% of the volume.

## Results

### The vials

**Fig. 3:**
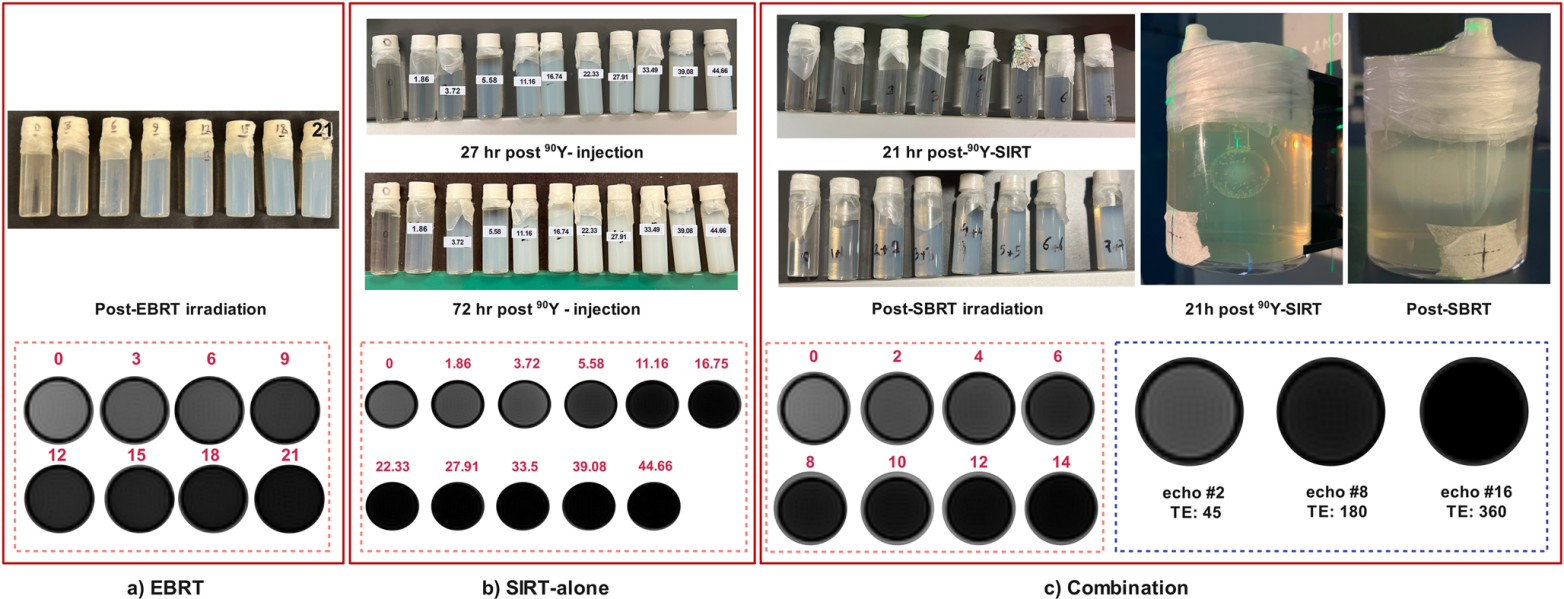
Representative vials irradiated with different absorbed doses along with an axial view of their corresponding MR images at echo#8 (TE = 180ms); (**a**) EBRT-alone vials (**b**) SIRT-alone vials after 27 and 72 h post SIRT, showing the color change of the gel proportional to the absorbed dose, and (**c**) combination therapy vials and phantom 21 h post SIRT and post SBRT. The blue panel shows the MR signal from a vial irradiated with 8 Gy (4 Gy SIRT + 4 Gy SBRT), displayed at three different echo times to illustrate MR signal decay behavior

Figure 3 shows the irradiated vials and the phantom after a certain amount of time post-irradiation. The MR images of the vials in this figure correspond to echo #8 (TE = 180 ms). The signal intensity (before R2 mapping) variation reflects changes in transverse relaxation due to different absorbed doses. The phantom irradiated with SIRT after 21 h and after SBRT irradiation is also included in this figure. As seen in the vial’s MR images, all samples regardless of irradiation type showed uniformity across the gel region, and an apparent slight ringing pattern within the gel region. The calibration vials for SIRT alone (Fig. 3b) and the combination treatment (Fig. 3c) exhibited areas of non-reactive gel to SIRT irradiation, likely due to Oxygen (O_2_) penetration from the lids. These regions were excluded from delineation and further analysis.

The added activity concentrations measured by the dose calibrator and PET, along with their corresponding absorbed doses for SIRT-alone and combination experiments, are presented in Table 3. The absorbed dose values calculated using PET-based activity measurements at filling time showed a mean relative error of −13.5% when compared to those measured by the dose calibrator. However, this study primarily focuses on dose values derived from dose calibrator measurements. It should be noted that direct measurements with the dose calibrator were performed only for the highest activity value for filling the calibration vials. The remaining activity concentration values were calculated relative to this reference value based on the volume of diluting gel added to prepare each vial dose. This approach was chosen to reduce measurement errors.

**Table 3:** The added activity concentrations for SIRT-alone and combination therapy experiments were measured by dose calibrator and PET images along with the calculated absorbed dose from MIRD in an ascending order. *The activities and dose values were corrected for injection time as PET images were acquired 13 h after filling the vials

	Vial Number	Activity concentration by dose calibrator (MBq/ml)	Activity concentration by PET at acquisition time (MBq/ml)	Activity by PET at filling time*(MBq/ml)	Dose by dose calibrator (Gy)	Dose by PET at filling time*(Gy)
^90^Y-SIRT-alone	1	0.073	0.024	0.028	1.860	0.747
	2	0.146	0.067	0.079	3.720	2.047
	3	0.220	0.106	0.124	5.580	3.235
	4	0.440	0.247	0.291	11.160	7.542
	5	0.660	0.432	0.507	16.750	13.155
	6	0.880	0.630	0.741	22.330	19.200
	7	1.100	0.754	0.886	27.910	22.959
	8	1.320	0.899	1.056	33.50	27.376
	9	1.540	1.147	1.348	39.080	34.946
	10	1.760	1.264	1.485	44.660	38.498
Combination	1	0.047	0.039	0.040	1.029	1.030
	2	0.095	0.079	0.082	2.059	2.087
	3	0.143	0.120	0.126	3.088	3.176
	4	0.190	0.159	0.167	4.118	4.211
	5	0.238	0.212	0.221	5.148	5.580
	6	0.286	0.252	0.264	6.178	6.640
	7	0.330	0.330	0.345	7.207	8.687

**Fig. 4:**
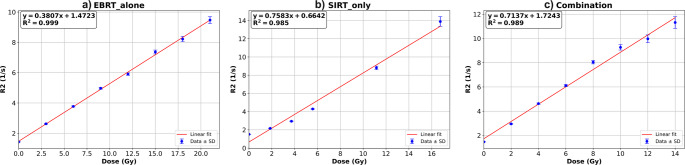
Dose response curves to irradiation from: (**a**) EBRT-alone, (**b**) SIRT-alone and (**c**) combination

A region approximately 2–3 pixels away from the vial’s walls (~ 1.5–2 mm) was excluded from the isotopically delineated areas. This was done to eliminate potentially unreliable dose-response data in peripheral regions caused by O_2_ penetration. Figure S2 illustrates the regions with O₂ inhibition effect and our approach of region selection to exclude these affected zones from further analysis. Figure 4 presents the final dose-response (calibration) curves derived from all irradiations. The gel exhibited a linear response to EBRT within the dose range of 0 to 21 Gy, with a sensitivity of 0.380 S^−1^. Gy^−1^, corresponding to the slope of the calibration curve.

For SIRT-alone irradiations, a linear relationship was observed within the 0–16.75 Gy range. The gel demonstrated saturation at doses exceeding this amount of absorbed dose, and as such, we excluded the corresponding datapoints from further analysis. For SIRT, the gel displayed a sensitivity of 0.758 S^−1^. Gy^−1^. In addition, a quadratic model was fitted to the data in the 0–16.75 Gy range of ^90^Y-SIRT and compared with the linear model using the Akaike Information Criterion (AIC) and F-statistical test. The quadratic model outperformed the linear model, with a lower AIC (−15.22 vs. −3.34) and a significant F-test (*p*-value = 0.014). The response curve with quadratic fit is shown in Figure S3.

The gel response to combination irradiations was linear in the 0–14 Gy range. The highest sensitivity to irradiation was observed in this type of irradiation (0.487 S^−1^. Gy^−1^). The mono-exponential curves showing the relationship between MRI signal vs. echo time for each irradiation are provided in Figures S4-S6. Uncertainty was assessed at multiple levels: the standard deviation of MR signals within each volume of interest (VOI), the 95% confidence intervals from exponential curve fitting, and the variability in R2 values used for dose–response modeling. Additionally, we refined VOI placement to exclude the inhomogeneous regions, resulting in improved consistency and accuracy in the final dosimetric results. The signals from first echo (TE = 22.5 ms) didn’t follow an exponential relationship, regardless of irradiation type, thus excluded from further evaluations. A figure showing the dose-response curve using all datapoints of ^90^Y-SIRT experiment is provided in supplementary section (Figure S7). Figure S8 shows the mono-exponential curves derived from all 16 points for combination therapy as an example.

### Phantom studies

**Fig. 5:**
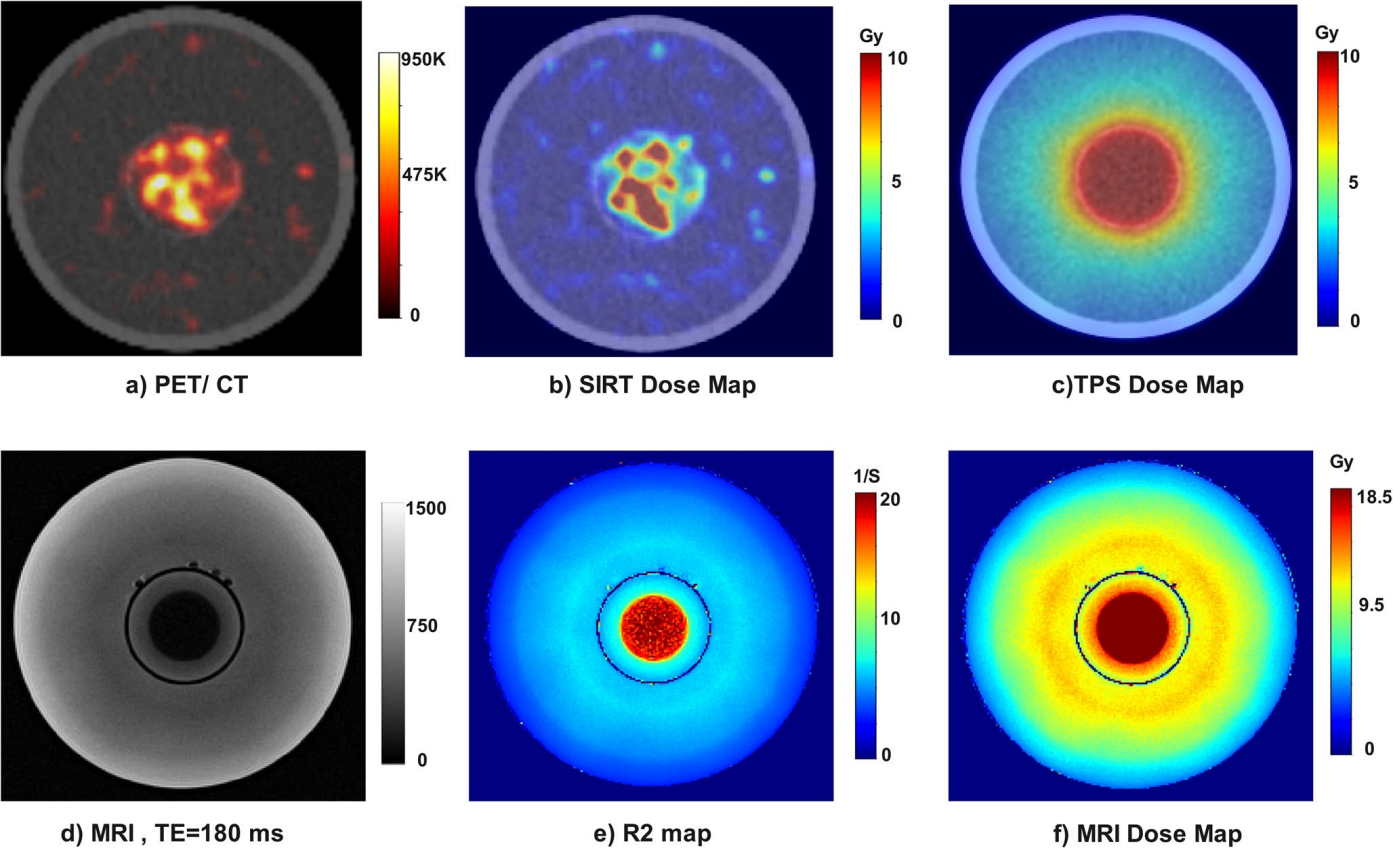
Representative images showing: (**a**) PET image from SIRT part of combination therapy overlaid on CT, (**b**) the dose map calculated from PET images based on the MIRD schema, (**c**) SBRT dose map extracted from treatment planning system (TPS), (**d**) MR image from the 8th echo (TE = 180ms), (**e**) R2 map calculated for MRI, and (**f**) the dose map of combination therapy calculated from R2 map

Our phantom was irradiated within the framework of combination therapy. Figure 5 depicts representative images including PET/CT, MRI and SIRT dose maps calculated from PET/CT images based on the MIRD schema, SBRT dose map from TPS, and combination R2 map and dose map from MR images, in an axial view at the center of the phantom.

A uniform dose map was expected to be induced from both SIRT and SBRT. For SBRT, a uniform dose map is observed. However, the SIRT dose map was not uniform due to colloidal trait of ^90^Y or likely an inherently non-uniform activity distribution. The HI and CV for tumor and WNL are listed in Table 4. The results indicate that the dose heterogeneity of ^90^Y is significantly higher than that of SBRT.

**Table 4:** The coefficient of variation (CV) and the heterogeneity index (HI) of absorbed dose within the tumor (sphere) and normal liver (cylinder excluding the sphere)

	HI-PET	HI-TPS	CV-PET	CV-TPS
Tumor	2.64	0.19	0.59	0.05
WNL	14.25	2.47	1.62	0.48

The MRI-based dose map from combination therapy exhibited a saturated area in the central core of the sphere and the peripheral region of the sphere with a diameter of ~ 4 mm showed an underestimated area.

The added activity concentrations and absorbed dose values (Gy) for SIRT, SBRT, and their combination are summarized in Table 5. According to results reported in this Table, the summation of the absorbed dose from PET and absorbed dose from SBRT (9.71 + 9.83) is ~ 19.54 Gy. However, the gel was able to measure 18.58 Gy, probably due to monomer depletion described in the Discussion section. The expected absorbed dose from SIRT within the cylinder was ~ 0.6 Gy, as measured by the dose calibrator. MIRD-based dosimetry using PET images showed good agreement with this estimate. However, following SBRT, the gel recorded an absorbed dose of 2.68 Gy, significantly higher than the combined expected dose from SIRT and SBRT (0.6 + 1.29 Gy). This discrepancy could partly be attributed to the limitations of the MIRD formalism, which is further discussed in the Discussion section.

**Table 5:** Summary of the injected activities to the different compartments of the Phantom and their corresponding dose values from different irradiation modalities. Note: the *±* standard deviations are derived from intra-volume dose heterogeneity

	Activity concentration by dose calibrator (MBq/ml)	Activity concentration by PET (MBq/ml)	Dose by dose calibrator (Gy)	Dose by PET (Gy)	Dose by SBRT (TPS, Gy)	Dose by MRI (gel, Gy)
Sphere (tumor)	0.38	0.36	9.99	9.71 ± 5.72	9.83 ± 0.45	18.58 ± 11.54
Cylinder (normal liver)	0.02	0.02	0.63	0.60 ± 0.98	1.29 ± 1.78	2.68 ± 1.82

**Fig. 6:**
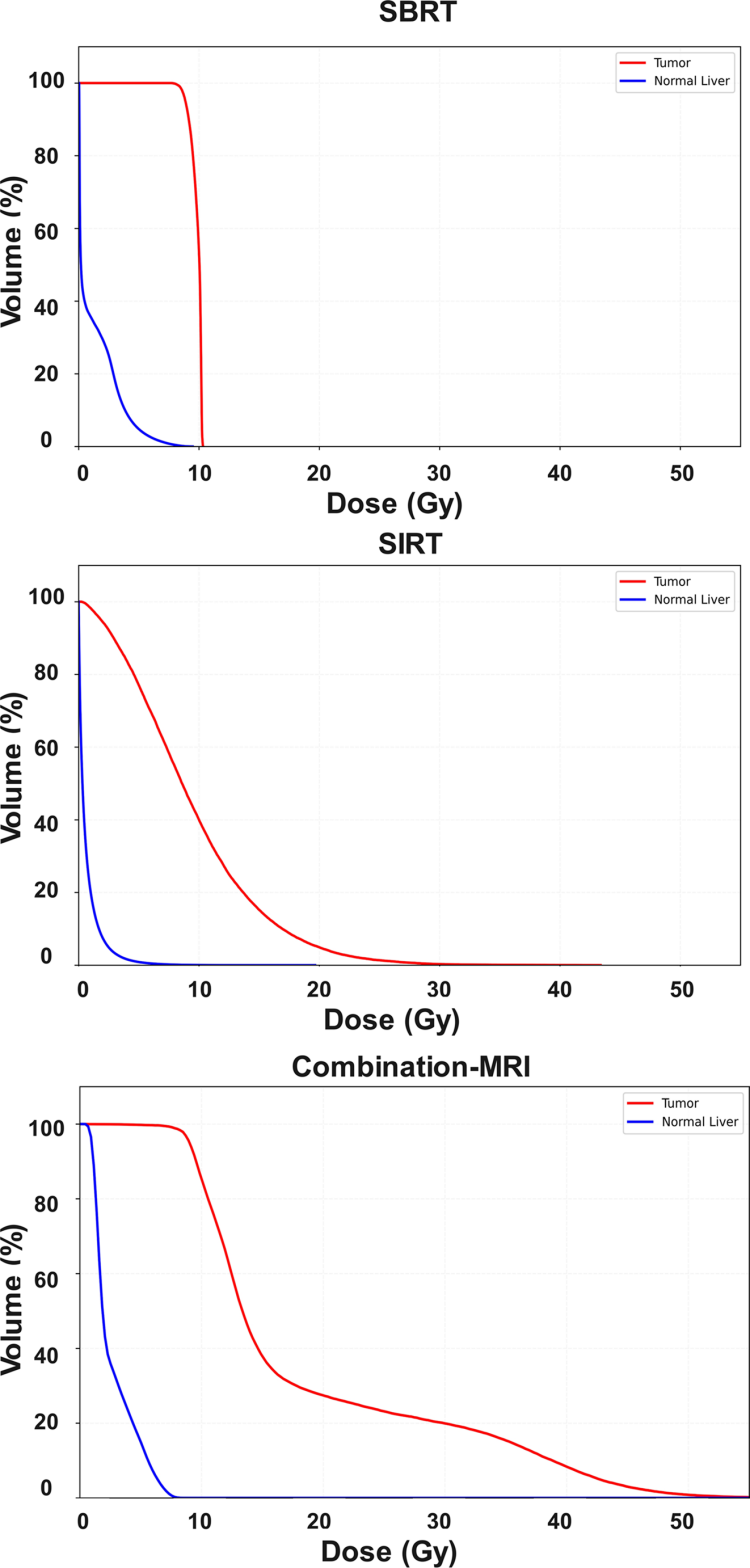
Dose-volume histograms (DVHs) from SBRT (TPS calculations), SIRT (PET-based dosimetry), and combination (MRI dose map)

Figure 6 shows the dose-volume histograms (DVHs) from SBRT (TPS dose map), SIRT (PET image), and combination (MRI dose map). The SBRT DVHs show a relatively uniform distribution. While SIRT DVHs for the sphere show a heterogeneous dose distribution, where different volumes receive varying doses meaning some regions of the volume are under/overdosed (the mean prescribed dose was 10 Gy). The combination indicates that the gel within both the sphere and cylinder (representing tumor and normal liver, respectively) successfully recorded the absorbed dose from both treatments. The cylinder (normal liver) DVH from combination therapy revealed higher absorbed dose and a larger area under the curve than might be expected from the sum of the SBRT and SIRT treatments.

**Fig. 7:**
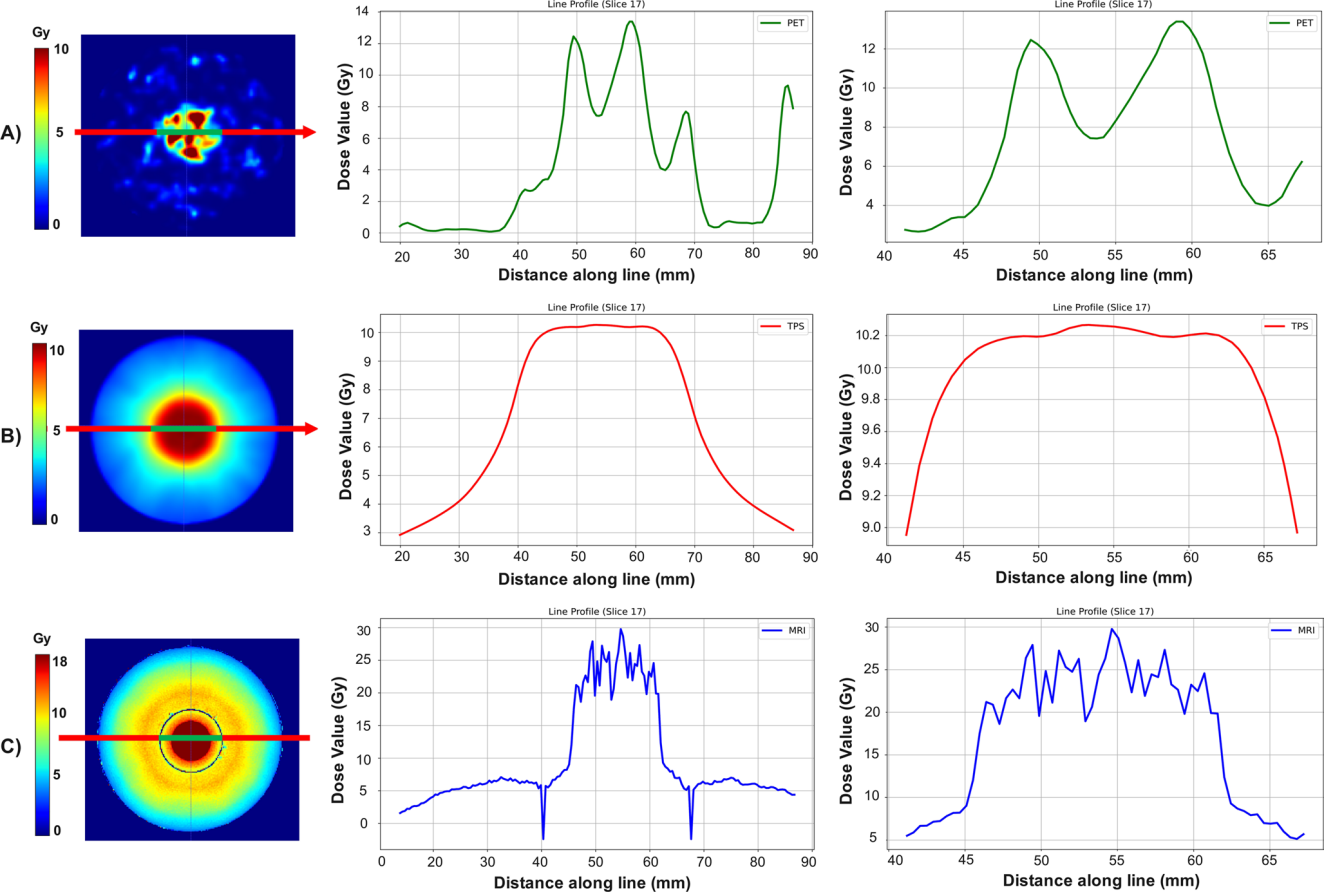
Line profiles taken across the horizontal view and central slice of the phantom. The guide shows the path along which the line profile is drawn within the cylinder (red) and sphere (green). Panel (**A**) shows the line profile from SIRT PET-based dose, (**B**) the SBRT dose, and (**C**) the combination dose. The line profiles are displayed for the entire line (middle column) and zoomed in to show the profile within the sphere (right column)

The line profiles along the central slice of the phantom including the sphere and cylinder from SIRT, SBRT and MRI results (combination) are shown in Fig. 7. The prescribed dose values were 10, 10, and 20 Gy for PET, TPS and MRI, respectively.

**Fig. 8:**
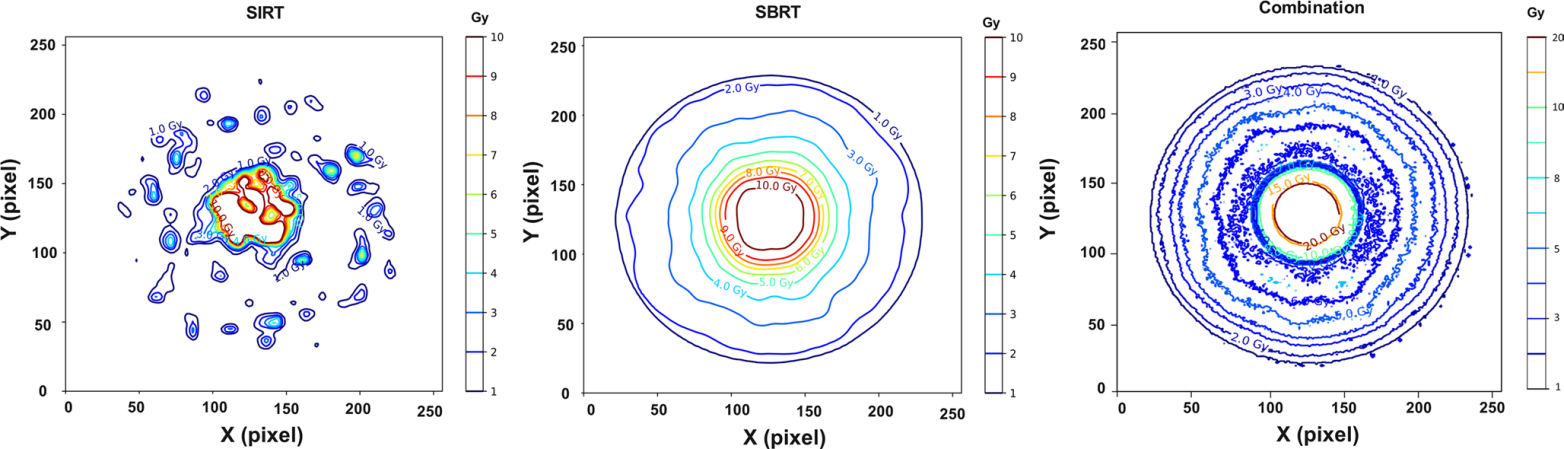
Isodose lines along the central axial slice of the phantom. Isodoses show a non-uniformity in SIRT (left) while a uniform absorbed dose is observed in SBRT (middle). The combination (right) of both was successfully recorded by MAGIC-f gel

The isodose lines (in Gy) from the axial central slice are presented in Fig. 8. The non-uniform distribution of the absorbed dose from ^90^Y-SIRT is evident, whereas SBRT shows a uniform dose distribution. For the combination therapy, the isodose lines demonstrate that the gel effectively captured the absorbed dose pattern from SBRT, along with an increase in dose values corresponding to the absorbed dose contributions from both treatments. The isodose lines appear thicker with a slightly noisier pattern probably due to 90Y distribution in an irregular pattern combined with SBRT. In addition, due to the process of R2 mapping on MR images and likely the presence of noise. We retained the original voxel resolution to preserve spatial fidelity. Future work may consider resampling MR images to reduce noise.

## Discussion

SIRT and SBRT are not biologically or effectively equivalent due to differences in modes of administration, dose rate, and other factors. Advances in SIRT dosimetry have facilitated its combination with SBRT. However, the current combination therapy approach relies on translating the biologically effective dose (BED) distribution of ^90^Y-SIRT into SBRT, using biological parameters (such as alpha/beta ratios) borrowed from EBRT for BED calculations [3, 5, 9].

The field is rapidly evolving, with efforts underway to develop dedicated biological factors for ^90^Y-SIRT using radiobiological modeling [10, 41]. Additionally, there have been attempts to use TOF PET-derived dose maps for guiding treatment planning in SBRT boost irradiation [4]. However, due to the highly precise spatial information required in SBRT, a significant challenge with this approach is aligning dose maps derived from TOF PET images, which have lower spatial resolution than planning CT or TPS dose resolutions. Consequently, this method may not be practical for small lesions (less than 2 cm³) and could miss some of the inhomogeneities in the ^90^Y-absorbed dose in larger lesions due to PET’s limited spatial resolution. Furthermore, there remains a lack of tools for direct measurement of absorbed doses without relying on SPECT- or PET-based dosimetry in the theranostics field.

Gel dosimetry is a valuable tool for recording 3D dose distributions with high spatial resolution. Its response to irradiation is considered independent of radiation energy and direction with a very small dependency to dose rate [33]. Additionally, it is tissue equivalent. Gel dosimeters come in various types, with polymer gel dosimeters (PGDs) being the most widely used for verifying complex therapy plans and quality assurance in EBRT. The mechanism of action of PGDs involves radiation-induced polymerization of monomers and cross-linking reactions within the gel matrix, which are proportional to the absorbed dose. This polymerization process results in changes to gel’s physical or optical properties, thereby enabling dose readout using various methods, including MRI (the gold standard), X-ray CT, optical CT, Raman spectroscopy, and ultrasound [12].

To contrast PET or SPECT/CT-based dosimetry and gel dosimetry advantages, we should compare common PET-based dosimetry limitations with read-out imaging modalities of the gel, particularly MRI as the gold standard. Due to substantial higher spatial resolution in MRI (submillimeter to 1 mm), the partial volume effect and the need for inhomogeneity correction is minimal with MRI. This holds even more promises with the optical-CT read-out method with higher spatial resolution and lower imaging time. In addition, gel dosimetry read-out is independent of radioactivity itself. The administered activity has already left its physical and chemical marks by changing the water mobility (and relaxation times), optical/mass density, acoustic impedance, etc. and does not need to be directly imaged. As a result, quantification is not affected by spill-in/out from neighboring high-activity or low-activity regions in the imaging sense. Moreover, when using gel dosimetry, we are not dealing with activity quantification (including attenuation and scatter correction) uncertainties, and SPECT/PET image artifacts. These are key advantages over PET or SPECT. However, this doesn’t imply that MRI or other gel readout imaging modalities are free from limitations. Longer acquisition time, magnetic artifacts and the need for multi-echo sequences can pose logistical challenges in MRI gel dosimetry.

MRI’s submillimeter spatial resolution (with an in-plane voxel size of 0.4336 × 0.4336 mm², in our study) enables accurate depiction of steep dose gradients between the tumor (sphere) and surrounding normal tissue (cylinder) and improves localization of high-dose regions.

Between 2005 and 2011, several studies explored the establishment and verification of polymer gel dosimetry for molecular radiotherapy using various radionuclides, including ^99m^Tc, ^32^P, and ^131^I [27–32]. In 2006, a research group demonstrated the feasibility and accuracy of the MAGIC gel dosimeter for ^32^P, showing good agreement with Monte Carlo simulations and highlighting its potential for 3D dosimetry [27, 30]. The same group conducted a phantom study in 2007 to evaluate MAGIC gel dosimetry, read out by MRI, for non-uniform distributions of ^131^I. The results were compared with SPECT-based dosimetry, and the study emphasized that gel dosimetry could serve as an independent benchmark for validating and improving patient-specific dosimetry [29]. This group further benchmarked the gel dosimeter against Monte Carlo methods in 2011 [28]. Additionally another type of gel dosimeter, named PAGAT, was investigated for use with ^99m^Tc [31]. Despite the promising early efforts, research activity in this area has declined. This is surprising given the unique advantages of polymer gel dosimeters in providing high-resolution, three-dimensional, and tissue-equivalent dose verification, especially in complex molecular radiotherapy settings.

Table 6 summarizes a selection of polymer gel dosimeters, emphasizing that while many other types exist, their application has primarily been in EBRT. To date, none has been validated for use in SIRT or in combination SIRT + SBRT therapies. This study is, to the best of our knowledge, the first to explore the feasibility of PGD for internal dosimetry applications in SIRT and combination of SIRT + SBRT. We also included bone-mimicking and deformable gels in the table, as these categories may hold promise for future applications in internal dosimetry, especially in anatomically complex or motion-sensitive contexts.

**Table 6:** Examples of different PGDs based on their composition, oxygen sensitivity, readout methods, and areas of application. The table includes both established and recent formulations, including gels designed for specific uses, such as bone-equivalent and deformable gels. While many of these gels have been successfully used in external beam radiotherapy (EBRT), their application in internal dosimetry (e.g., SIRT) or combination therapies (e.g., SIRT + SBRT) remains limited or unexplored. PAGAT: polyacrylamide agarose gelatin fabricated at atmospheric conditions, PAG: polyacrylamide agarose gelatin, VIPAR: N-vinylpyrrolidone argon, DMSO: dimethyl sulfoxide

Gel Dosimeter	Monomer Type	Oxygen Sensitivity	Readout Method	EBRT/Brachytherapy	Internal dosimetry	Special Feature
MAGIC	Methacrylic acid	Low (normoxic)	MRI	Yes [42]	Limited (^131^I [28, 29, 32], ^32^P [27])	Tissue equivalent
MAGIC-f	Methacrylic acid	Low (normoxic)	MRI	Yes [33, 34]	^131^I [43], & ^90^Y-SIRT (our study)	Dose-response stability, High melting point
PAGAT	Acrylamide	Moderate (normoxic)	MRI, Optical CT	Yes [44]	Limited(^99m^Tc) [31]	Good sensitivity
PAG	Acrylamide	High (anaerobic)	MRI	^90^Sr/^90^Y intravascular brachytherapy [45]	-	First widely studied polymer gel dosimeter
VIPAR	Acrylamide	High (anaerobic)	MRI	Yes [46]	-	Wide dynamic dose range
Bone-mimicking Gel	Variable (with Ca or hydroxyapatite)	Variable	MRI, CT	Yes [47]	-	Simulates bone density
Deformable Gels	Varies (e.g., VIPAR + DMSO)	High or normoxic	Optical CT	Yes [48]	-	Adaptive RT, motion studies

MAGIC-f is a PGD whose characteristics were extensively evaluated for EBRT [33]. However, it has not been studied for internal dosimetry until now. To the best of our knowledge, this is the first instance of using this gel to record the 3D dose distribution from therapeutic radionuclides such as ^90^Y.

Our results showed that the gel’s response to EBRT and combination irradiations was linear within the range of absorbed doses studied. However, the linear dynamic range for the SIRT-alone study was found to be between 0 and 16.75 Gy. The gel response was saturated/nonlinear for vials with larger absorbed doses. This saturation may be attributed to the depletion of monomers within the limited volume of the calibration vial, as well as the prolonged irradiation time required due to the low dose rate of ^90^Y. The impact of such low dose rates on the gel’s response warrants furthur detailed investigation in future studies.

Based on the vial volume used, the color changes and contrast of the vials became visually evident around 24–28 h post-SIRT. Although in this study, MRI readout was performed after approximately one half-life of ^90^Y to ensure stability and to observe visible color changes in the gel, this timing is not strictly required. Earlier imaging is feasible as polymerization-induced changes in magnetic resonance properties can be detectable before color changes are visible. However, gel dosimeters are passive dosimeters and cannot be used for real-time dosimetry. This limitation is due to the need for post-irradiation polymerization, a stabilization period, and the time required for readout. Several studies have investigated possible strategies to reduce this latency [49]. For MAGIC-f PGD in particular, these include optimizing gel formulation to accelerate polymerization [33], employing fast MRI sequences or alternative imaging techniques, such as rapid optical CT scanning [50], incorporating nanoparticles, such as gold nanoparticles into the gel matrix, to act as catalysts or enhance MRI contrast [51].

We acknowledge that the local energy deposition model assumes voxel-level self-calibration and does not explicitly account for beta particle range. Given the lack of tissue heterogeneities in our setup, the impact of neglecting cross-voxel contributions is expected to be minimal. Moreover, the local energy deposition model reflects standard practice in routine ^90^Y dosimetry in many clinical and research centers. Future studies should consider validation against Monte Carlo-based dosimetry.

The activity measurements for ^90^Y should be carefully implemented. A discrepancy of −13.5% was observed between PET-derived and dose calibrator-measured activities. This difference may arise from several factors associated with both modalities, as well as volume measurement uncertainties when using syringes to pick up a certain amount of gel from the main beaker. Accurate assay of ^90^Y, a pure β⁻ emitter, using a dose calibrator is inherently challenging. The measurement relies on indirect detection of Bremsstrahlung photons, which is highly sensitive to source geometry, matrix composition, and surrounding materials [52, 53]. In addition, the colloidal formulations of ^90^Y-citrate, responsible for non-uniform distribution within the volume, may further exacerbate measurement variability even in case of exact extraction of the calculated volume when diluting the activities. On the PET side, quantification from VOIs drawn on the calibration vial may be affected by partial volume effects. Furthermore, even though dose calibrators are routinely used in clinical setting, their accuracy and reproducibility are within a range of uncertainty. A sensitivity analysis or Monte Carlo simulation could address this in future studies.

We measured the activity only once for the vials containing the highest activity and prepared the remaining vials by diluting the gel for lower activities. Each time we added non-irradiated gel to the irradiated batches, we manipulated with care to ensure uniform mixing of the activity with the gel, considering the colloidal nature of ^90^Y-citrate. Without proper mixing, sampling with syringes, pipettes, or other tools could lead to inaccuracies, as the sample could come from regions of the batch with higher or lower concentrations of ^90^Y. Speaking of colloidal nature of ^90^Y, both visual assessment and quantitative analysis of dose inhomogeneity within the phantom, compared to SBRT (delivered with a uniform dose distribution), demonstrated that ^90^Y-SIRT contributes significantly to absorbed dose and spatial heterogeneity.

The sensitivity of the gels was different across the experiments as the gels were prepared in different batches on different days of experiments. The ringing artifacts (A.K.A Gibbs artifact or truncation artifact) within the vials occurred due to limitations in spatial resolution and signal processing. However, these artifacts didn’t affect the R2 mapping and dosimetry results. The oxygen penetration effect was not directly evaluated in this study; however, the regions of the vials and phantom that were susceptible to O_2_ penetration such as the regions close to the lids or edges were excluded from further analysis to cope with oxygen contamination issue. In MR images of vials, the first echo time did not follow the expected exponential curve due to susceptibility to errors caused by ​B1 inhomogeneities and imperfect refocusing pulses, which is a common issue. To address this, the first echo was discarded from the analysis for both vials and phantoms to correct for related artifacts. However, the effect of removing the first echo on artifact correction was not specifically studied in this work [40].

In the combination experiment, the absorbed dose values were carefully selected based on our experience with SIRT-alone and EBRT-alone experiments to ensure they fall within the linear response range of the gel dosimeter for both modalities and did not reach a saturation point before MR imaging. Although the phantom absorbed doses did not exceed 14 Gy from both modalities, a wider calibration range in ^90^Y-SIRT experiment was necessary to identify the gel’s linear response region, which was extended up to ~ 16.5 Gy.

In the combination experiment with the phantom, the peripheral region of the sphere (approximately 4 mm in diameter) showed an underestimation of absorbed dose values. This underestimation may have resulted from monomer depletion (the reduction in the concentration of available monomers as they undergo polymerization when exposed to radiation) in the central area and caused a saturation. Polymerization likely began in the central part of the sphere first. However, due to prolonged irradiation, fresh monomers from the peripheral zone diffused into high-dose regions (central regions) and kept polymerizing, making the gel showing a higher dose than was actually delivered locally. As a result, the central core became saturated and consumed the majority of the available monomers before polymerization could occur in the periphery. This phenomenon has been well-described in a study by Dedeen et al. on gel dosimetry for brachytherapy with a ^192^Iridium source [54].

In contrast, the absorbed dose values inside the cylinder were also higher than expected from the recovered PET-based and SBRT absorbed dose values together. Such a discrepancy is likely due to the fact that the MIRD dosimetry approach for beta emitters does not account for internal bremsstrahlung and other types of radiation, such as annihilation photons. Recent studies have highlighted that the effect of internal bremsstrahlung in dosimetry and radioprotection modeling of pure beta emitters is often disregarded and considered negligible, and it is typically excluded from dosimetry calculations, even in Monte Carlo simulations [55, 56]. However, this omission overlooks its contribution to the total absorbed dose and should be carefully addressed [56]. Auditore et al. reported that dose point kernels (DPKs) commonly used for beta emitters neglect continuous photon emission from internal bremsstrahlung, resulting in a 20–34% underestimation of the absorbed dose [55]. However, in this study, apparently the gel dosimeter was able to record the dose contributions from all radiation components, leading to overestimated dose values within the cylinder. In this regard, the results of summing or subtracting the three modalities from each other did not reproduce the outcomes of each dosimetry approach individually. Gamma evaluations showed the same pattern when comparing each pair of the dose maps, this is probably because each time the test had compared a combination of the same three modalities.

The CT scans from PET/CT post-SIRT, KVCT, and CBCT during SBRT undoubtedly contributed absorbed doses to the gel. The absorbed dose from these modalities can be calculated using the reported CTDI_vol_ values. However, these doses are negligible (as a rule of thumb less than 50 cGy from all CTs) compared to the absorbed dose values from SIRT or SBRT themselves, that were not calculated in this study. Nevertheless, the CT scans were essential for accurately locating the dose maps. We used CT- and MRI-compatible point markers to locate the dose maps and enable comparison between corresponding regions.

The results of this study showed the feasibility and limitations of combination therapy dosimetry using MAGIC-f PGD. By considering the privileges and limitations, we plan to conduct a Monte Carlo study for both activities and absorbed dose calculations to further evaluate the gel’s performance in the future. Additionally, we intend to perform MR imaging post-SIRT and use MRI-based dose images as a guide for SBRT treatment planning. While we initially attempted to use the calculated dose maps from PET/CT for this purpose, last-minute unexpected incompatibilities between the TPS and the format of our dose maps prevented their use. As a result, we had to resort to a uniform SBRT irradiation due to this technical issue. One important feature for developing any commercial radionuclide therapy dosimetry software is enabling exportation and compatibility of the dose map with EBRT TPS for this matter [57].

The vials used for SIRT and combination therapy could have been larger to avoid saturation caused by monomer depletion. While we demonstrated the feasibility of combination therapy using PGDs, a more realistic mimic of body anatomy could be achieved using a more elaborate anthropomorphic phantom. For further evaluations, a 3D-printed phantom could be designed to replicate the liver within the body. However, the compatibility of the 3D printing material with the gel’s response must be carefully considered [58].

We believe that gel dosimetry holds great potential as a tool for 3D dosimetry in theranostics. Our findings demonstrate that the absorbed dose inhomogeneities in SIRT, as well as its combination with SBRT, can be visualized with greater spatial resolution than traditional PET/CT imaging. In addition to radionuclide therapy with ^90^Y, gel dosimetry facilitates pre-therapeutic dosimetry for other radionuclides and radiopharmaceuticals used in theranostics, such as ^177^Lu. For alpha emitters like ^225^Ac, where visualizing radionuclide distribution and dosimetry is limited by current medical imaging techniques, gel dosimetry offers a promising solution for visualization of the “unseen” with MRI’s high spatial resolution. Given the challenges of MRI cost, artifacts, and time-consuming readouts, gel dosimetry can be effectively performed with alternative readout systems, such as X-ray CT or dedicated optical CT, making it a feasible and commonly used approach in theranostic centers.

## Conclusion

The MAGIC-f polymer gel dosimeter has demonstrated promising results in capturing dose distributions from three different irradiation techniques; EBRT, ^90^Y-SIRT, and a combination of SIRT and SBRT. Inhomogeneities in absorbed dose distributions were captured with greater spatial resolution than PET/CT, making this tool a potentially more effective guide for SBRT boost treatment planning verification in combination therapy. The presented method might be promising in practical 3D dosimetry for various therapeutic radioligands, potentially for use alpha-emitting radionuclides.

## Electronic supplementary material

Below is the link to the electronic supplementary material.

Supplementary Material 1

## Data Availability

The data used in this work is not available.

## Abbreviations

3D: three dimensional
AAA: Anisotropic Analytical Algorithm
BED: biologically effective dose
CT: computed tomography
CTDI_vol_: computed tomography dose index volume
DICOM: digital imaging and communications in medicine
DD: dose difference
DTA: distance to agreement
DMSO: dimethyl sulfoxide
DVH: dose-volume histogram
EBRT: external beam radiation therapy
FFF: flattening filter free
FOV: field of view
FWHM: full-width at half maximum
HPLC: high-performance liquid chromatography
MAGIC-f: methacrylic and acrylamide-based gel initiated by copper with formaldehyde
MIRD: medical internal radiation dose
MRI: magnetic resonance imaging
NEX: number of excitations
OSEM: ordered subset expectation maximization
PAGAT: Polyacrylamide agarose gelatin
PET: photon emission tomography
PGD: polymer gel dosimeter
PMMA: polymethyl methacrylate
PSF: point spread function
PTV: planning target volume
SBRT: stereotactic body radiation therapy
SIRT: selective internal radiation therapy
SPECT: single photon emission tomography
SSD: source-to-skin distance
TCP: tumor control probability
TE: echo time
TOF: time of flight
TPS: treatment planning system
TR: time of repetition
VIPAR: N-vinylpyrrolidone argon
VMAT: volumetric modulated arc therapy
VOI: volume of interest

## Code Availability

The code developed in this work is available upon request.
