# Supplementary material for: Evaluation of end-to-end 3D absorbed dose distribution in 90Y-SIRT and SBRT combination therapy using MAGIC-f polymer gel dosimeter
Source: Eur J Nucl Med Mol Imaging. 2025 Aug 8;53(2):1313–30. doi: 10.1007/s00259-025-07461-2 (PMC12830405; doi:10.1007/s00259-025-07461-2)
Supplement: Supplementary file 1 — Supplementary Material 1 [file 259_2025_7461_MOESM1_ESM.pdf]

Supplemental material

Materials and Methods

Planning CT from vials and phantom prior SBRT

Planning CT was conducted using the Siemens SOMATOM go.Sim (Siemens Healthineers, Erlangen, Germany). The phantom was filled with room-temperature distilled water and positioned horizontally on its holder, then aligned using laser guidance. Three metal spot markers were placed, two laterally at one end of the cylinder and one on the other end, to ensure they were distant from the sphere, preventing any metal artifacts that could affect dosimetry calculations.

For calibration purposes, vials were placed in a plexiglass vial holder that was positioned inside a plexiglass box tank (25 × 25 × 25 cm) filled with room-temperature water, with 7 cm of water above the vial caps to provide sufficient scattering material. The rack was secured to one side of the tank to ensure reproducible positioning during the irradiation session. The center of the vials was 5 cm from the outer wall of the tank.

Planning CT images of the phantom and the vials were acquired separately using an abdominal protocol with settings of 120 kVp and a tube current of 13 mA. A slice thickness of 1 mm was used for the phantom to achieve the necessary spatial resolution. For the vials, a 2 mm slice thickness was selected to achieve a compromise between imaging time and resolution, as the focus was on a homogeneous dose calculation.

$SIRT_{alone}$ Irradiation

We considered radioactive concentrations corresponding to dose values starting from ~ 45 Gy decreasing to 1 Gy in eleven vials.

Given the low activity levels involved (see Table 3), and errors related to ^{90}Y activity measurements using the dose calibrator, precise activity dispensing was critical. Therefore, we proposed and implemented an approach to minimize the errors related to activity assaying, as much as possible.

295.14 mL of the total gel (300 mL) prepared for this experiment was allocated for calibration vial preparation. This volume was further divided into batch#1 (49.19 mL gel) and batch#2 (245.95 mL gel) in two beakers. The activity required to deliver an absorbed dose of 44.66 Gy to the first vial was calculated (44.57 MBq). This corresponded to 0.81 mL of 90Y-Citrate. The net measured activity (44.57 MBq) was added to batch#1 (49.19 mL gel). Then the solution was mixed thoroughly using a magnetic stirrer for at least 10 minutes. 10 mL of this radioactive mixture (gel+^{90}Y), was transferred into the first calibration vial using a syringe, filling the vial completely.

To prepare the rest of calibration vials we used batch#2. First, we added 4.05 mL of high-performance liquid chromatography (HPLC-grade) water to Batch#2 and thoroughly mixed using a magnetic stirrer. This was done to compensate for the 2% water initially removed during gel preparation. Then, 5.71 mL of the gel from this batch was pipetted into the remaining gel in radioactive batch (batch #1) to create a solution with 39.08 Gy absorbed dose. The solution was mixed for 10 min.Then, 10 ml of this mixture was collected to fill the corresponding vial. This process of stepwise dilution and volume dispensing was repeated to prepare subsequent vials corresponding to absorbed doses down to 1.86 Gy. The amount of gel for dilution of the radioactive batch (batch#1) to reach 33.5, 27.91, 22.33, 16.75, 11.16, 5.58, 3.72, and 1.86 Gy were 5.95, 6.33, 7, 8.33, 11.67, 25, 20, and 50-ml gel, respectively. The vials were kept shielded in the fridge before SPECT/CT, PET/CT and MR imaging. The shielding used included a lead pot with a thickness of 1 cm for vials and lead blocks with a thickness of 5 cm for the phantom. Activity measurements were conducted using a well-type ionization chamber (MED ISOMED, Model 2000/2010, calibrated for ^{90}Y

measurements and verified annually). Any residual radioactive gel remaining in the syringe after gel injection was not reused and was disposed as radioactive waste, in accordance with radiation safety protocols Moreover, we used a separate syringe for filling each vial to ensure accurate solution concentrations and avoiding contamination.

$SIRT_{alone}$: PET/CT imaging

The calibration vials underwent PET/CT imaging prior to MRI readout, The PET/CT scan was conducted on the Siemens Biograph Vision.X (Siemens Healthineers, Erlangen, Germany) at Geneva University Hospital after the samples were in the fridge (4° C) for 13 hours. A clinical routine imaging protocol for SIRT in our center was followed, with a scan duration of 30 minutes. Image reconstruction was performed using a 3D iterative ordered subset expectation maximization (OSEM) algorithm with 5 iterations and 5 subsets, incorporating resolution recovery and time of flight (PSF + TOF), and a post-reconstruction Gaussian filter with a 2-mm full-width at half maximum (FWHM). The reconstructed matrix size was 440 × 440, with a slice thickness of 1.4 mm and pixel spacing of 0.825 × 0.825 mm. Scatter and attenuation corrections were applied using model-based relative scatter modeling and low-dose CT for attenuation correction. The low-dose CT scans were acquired at 80 kVp, with a tube current of 64 mA, a pitch factor of 0.8, and a slice thickness of 1 mm. The computed tomography dose index volume ($CTDI_{vol}$) was 0.74 mGy.

Combination therapy

This part is explained in two different sections "Combination therapy: ^{90}Y-SIRT", and "Combination therapy: $SBRT_{boost}$" to complete the Methods section

Combination therapy: ^{90}Y-SIRT

Like $SIRT_{alone}$, 300 ml gel, was separated to fill the calibration vials. First, we further divided this amount of gel into two batches: batch#1, a beaker containing 60 ml of gel to prepare the vial with the highest absorbed dose value (7Gy), and batch #2 which contained 240 ml gel, diluted by adding 3.89 ml of HPLC grade water to compensate for the initial reduction of water in the gel compositon.

A total of 16.541 MBq in 0.97 ml of ^{90}Y-Citrate solution was injected into batch#1 to create 7 Gy of absorbed dose within the vial after 68h. The solution was mixed up for at least 10 minutes using a magnetic stirrer to get a homogenous irradiated sample. The first vial was filled with gel to create 7 Gy. Then we diluted the remaining gel in batch #1 by adding gel from batch #2 to create the other vials. To this end, we used 11.85, 10.37, 13.06, 18.43, 31.85, and 85.56 ml of unirradiated gel from batch #2 to create the vials with absorbed doses of 6, 5, 4, 3, 2, 1 Gy, respectively. After every step, the solutions were mixed up homogeneously for 10 mins before filling the vials, and the syringes used for filling each vial were thrown out and never used to fill other vials.

In the next step, the remaining 550 ml of the total gel was used to fill the phantom. We again separated two batches: batch #1 (49.19 ml) to prepare the radioactive gel for filling the sphere, and batch #2 (491.89 ml) for the cylinder. The goal was for the sphere to receive 10 Gy, and the cylinder 0.5 Gy of absorbed dose after 68 hr. To this end, 19.106 MBq of ^{90}Y-citrate (in 0.81 ml) was mixed with batch #1, and then the mixture was used to fill the sphere. Similarly, 9.648 MBq of ^{90}Y-citrate (in a volume of 8.11 ml) was added to batch #2, and the mixture was used to fill the cylinder. The mixtures were thoroughly stirred for 20 minutes before being poured into the sphere and the cylinder. The samples were shielded and stored in a refrigerator for 22 hours before undergoing SBRT irradiation. This time was required for gelation and to observe the gel's color change due to SIRT irradiation prior to initiating SBRT.

We relied on data from dose calibrator as the reference instead of PET. The measured radioactivity concentrations were 0.019 MBq/mL I the cylinder and 0.388 MBq/mL in the sphere. based on these measurements and dose estimations using the MIRD formalism, the calculated absorbed doses were 0.637 Gy for the cylinder and 9.999 Gy for the sphere.

Combination therapy: $\mathbf{SBRT_{boost}}$

The phantom was positioned precisely in the center of the holder, so the sphere remained away from the edges of the holder, consistent with the CT simulation setup. Orthogonal X-ray kV imaging was performed at 100 kV and 2.56 mAs to facilitate quick positional adjustments. Immediately before radiation delivery, a 3D CBCT scan was acquired at 100 kVp and 50 mAs and matched to the CT simulation images to allow for real-time adjustments by modifying the table position in 6 dimensions. This step is critical in SBRT, where even minor deviations can significantly affect dose delivery due to the high level of precision required. CT simulation, kV imaging, CBCT, were extracted as DICOM files for further analysis.

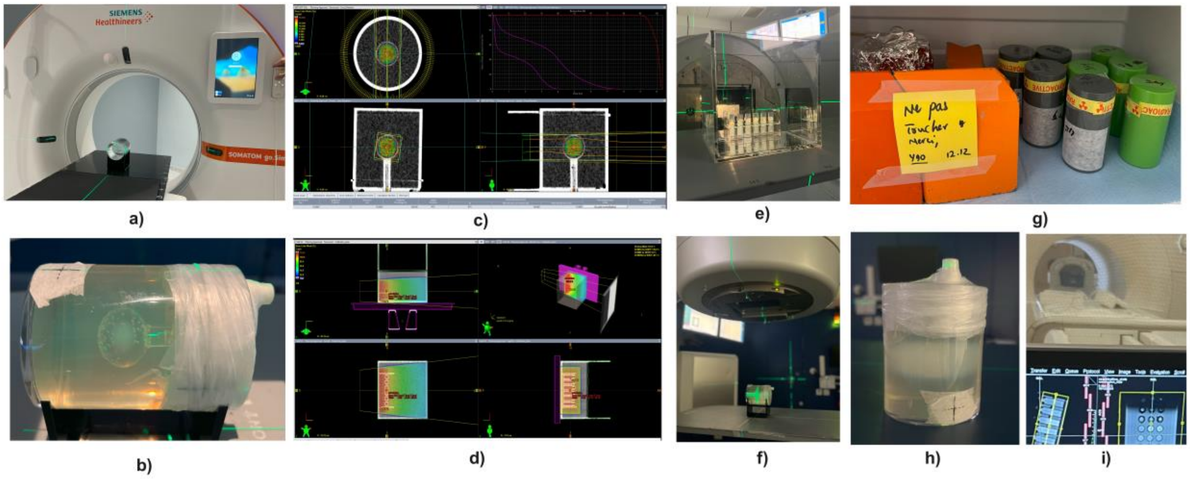

Figure S1. Shows the whole procedures of combination therapy, a) CT simulation of the phantom b) phantom 21 post-SIRT, c) treatment planning for SBRT, d) treatment planning for the vials, e) vials Irradiation, f) phantom SBRT Irradiation, g) storing of the vials and phantom with shielding in the fridge, h) phantom post SBRT and SIRT, and i) MRI.

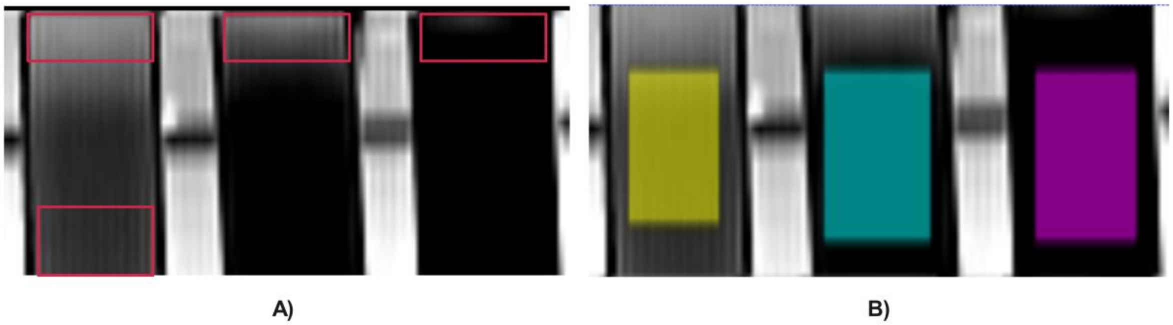

Figure S2. (A) Vials from the SIRT-only experiment. The red-marked regions indicate areas with significantly deviated MR signals, likely due to oxygen penetration and proximity to the vial walls. B) VOIs used for the analysis, positioned centrally to avoid inhomogeneous signal regions and ensure more reliable quantification.

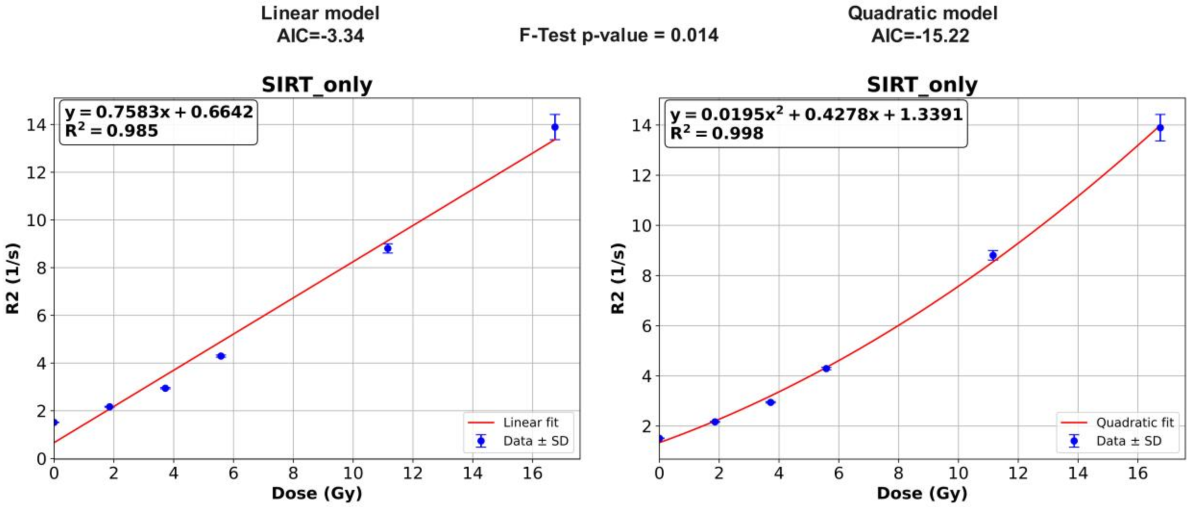

Figure S3. Comparison between linear and quadratic models of dose response curve (^{90}Y-SIRT).

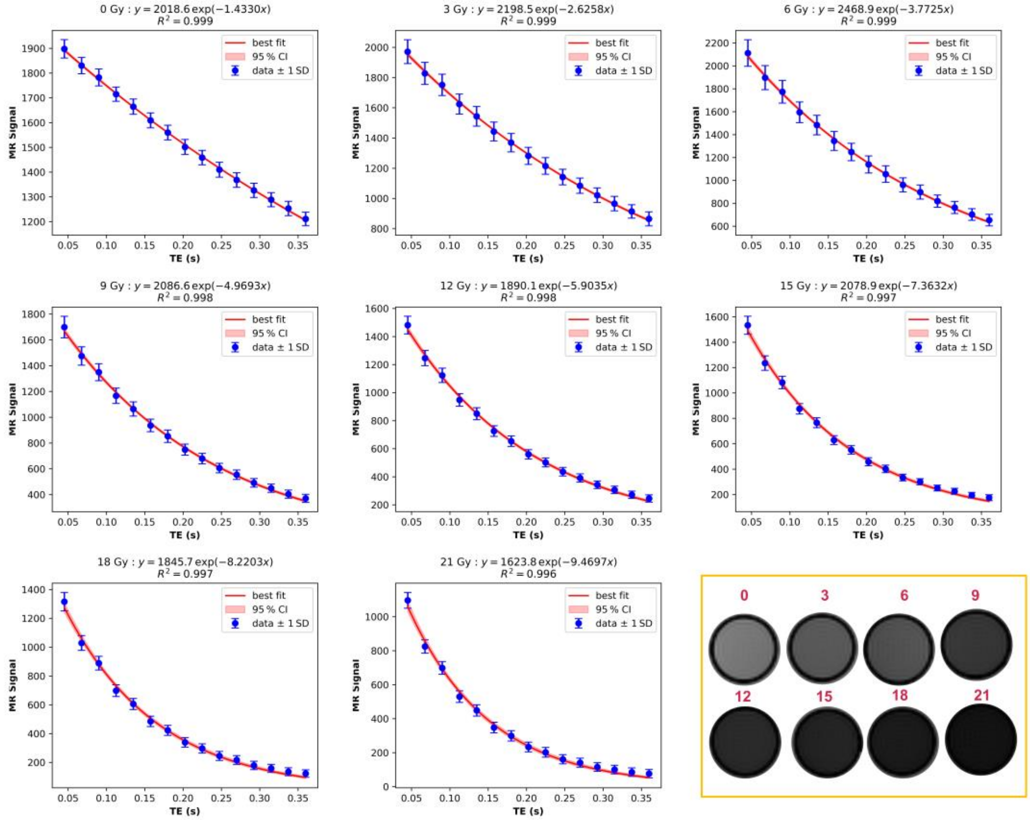

Figure S4. The mono-exponential curves demonstrate the relationship between MR signals and last 15 echo times(s) for ***EBRT-alone*** irradiations for different absorbed doses. The R2 (1/s) were measured from these curves. The error bars represent the standard deviation of MR signals collected from a VOI in the vial MR images. The red shaded area represents the 95% confidence interval (CI) of all possible exponential fits to the data points.

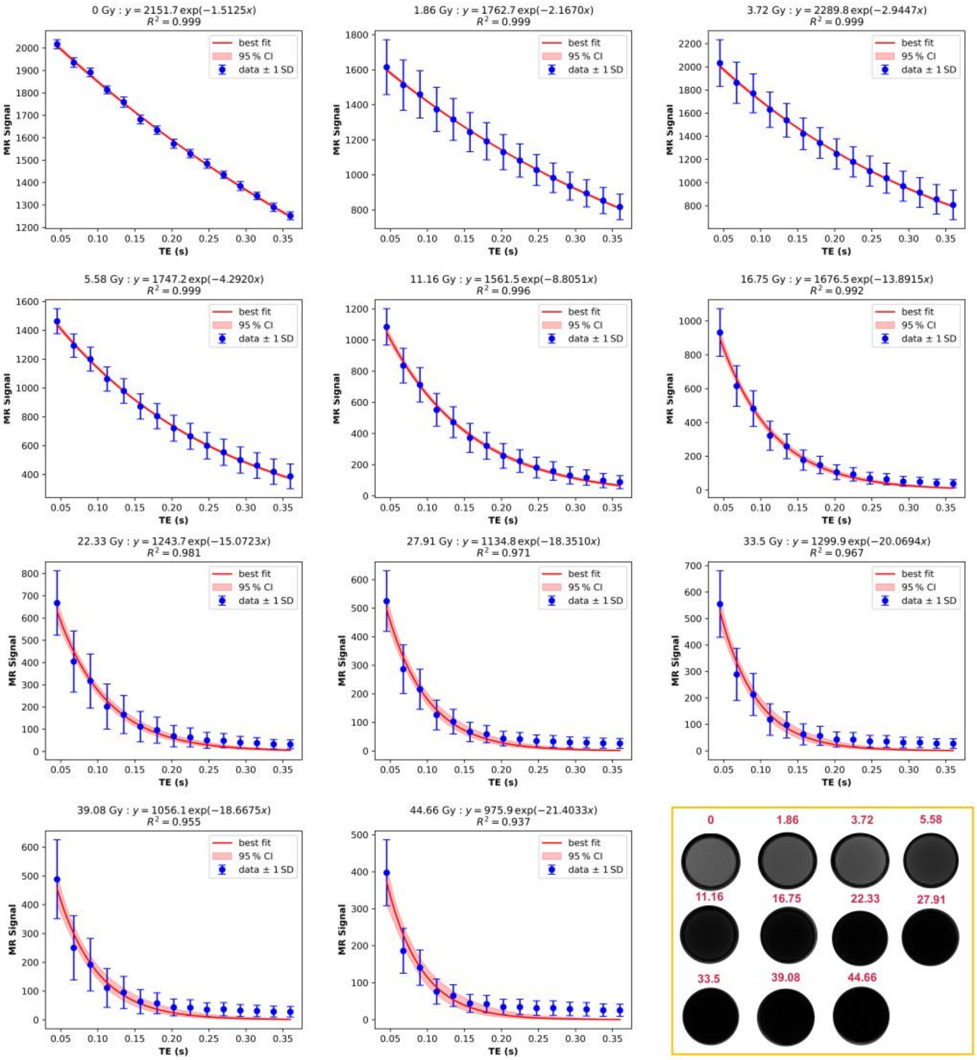

Figure S5. The mono-exponential curves demonstrate the relationship between MR signals and last 15 echo times (s) for ***SIRT-alone*** irradiations for different absorbed doses. The R2 (1/s) were measured from these curves. The error bars represent the standard deviation of MR signals collected from a VOI in the vial MR images. The red shaded area represents the 95% confidence interval (CI) of all possible exponential fits to the data points.

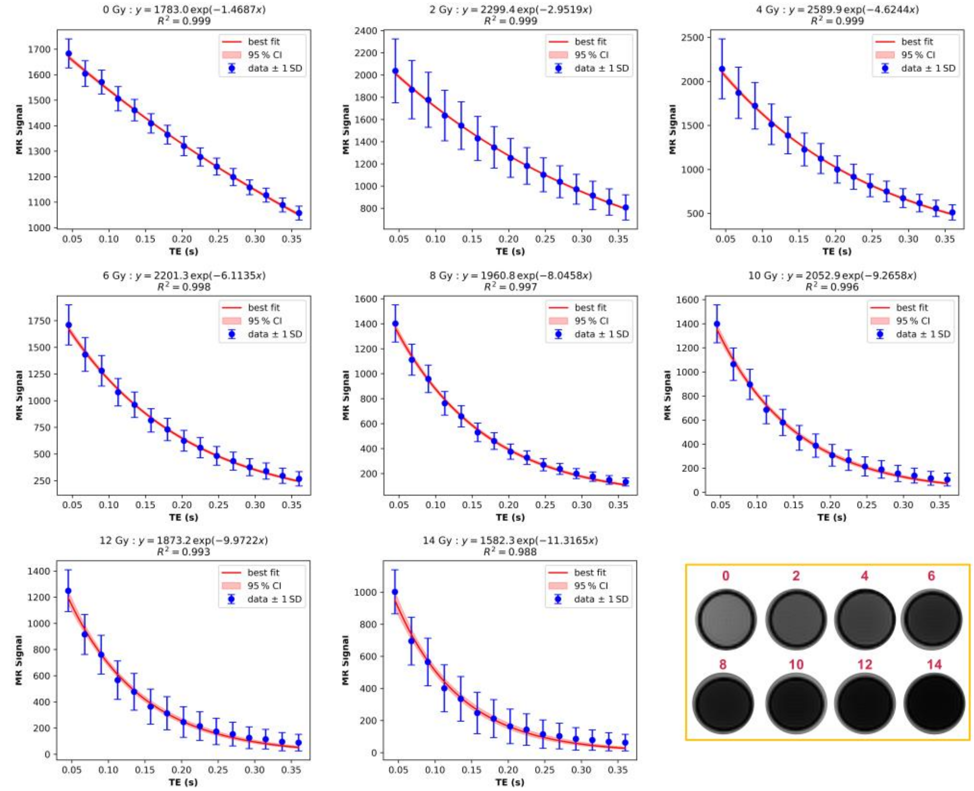

Figure S6. The mono-exponential curves demonstrate the relationship between MR signals and the last 15 echo times (s) for ***combination*** irradiations for different absorbed doses. The R2 (1/s) were measured from these curves. The error bars represent the standard deviation of MR signals collected from a VOI in the vial MR images. The red shaded area represents the 95% confidence interval (CI) of all possible exponential fits to the data points.

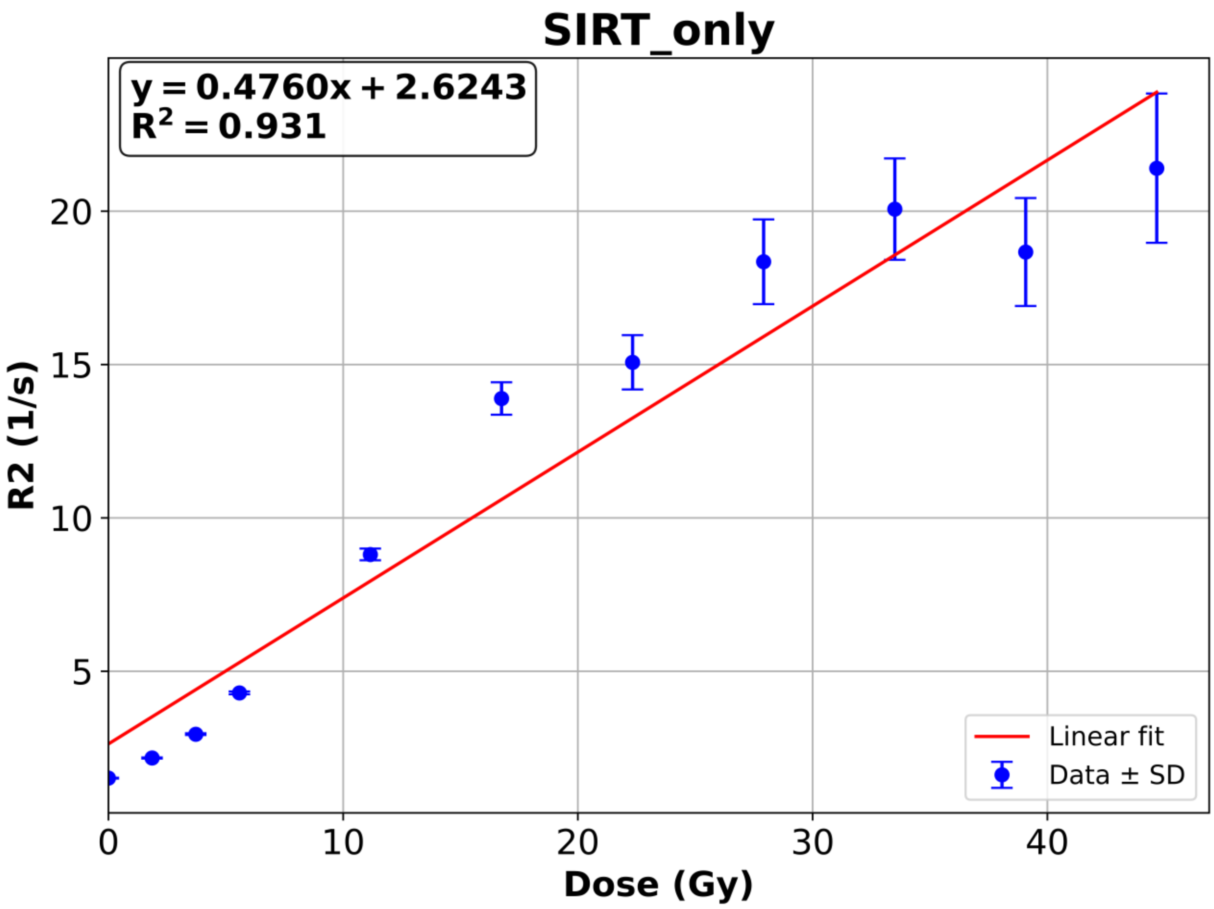

Figure S7. The dose-response curve for 90Y-SIRT-alone experiment using the entire datapoints. The gel showed a linear response between 0-16.75 Gy.

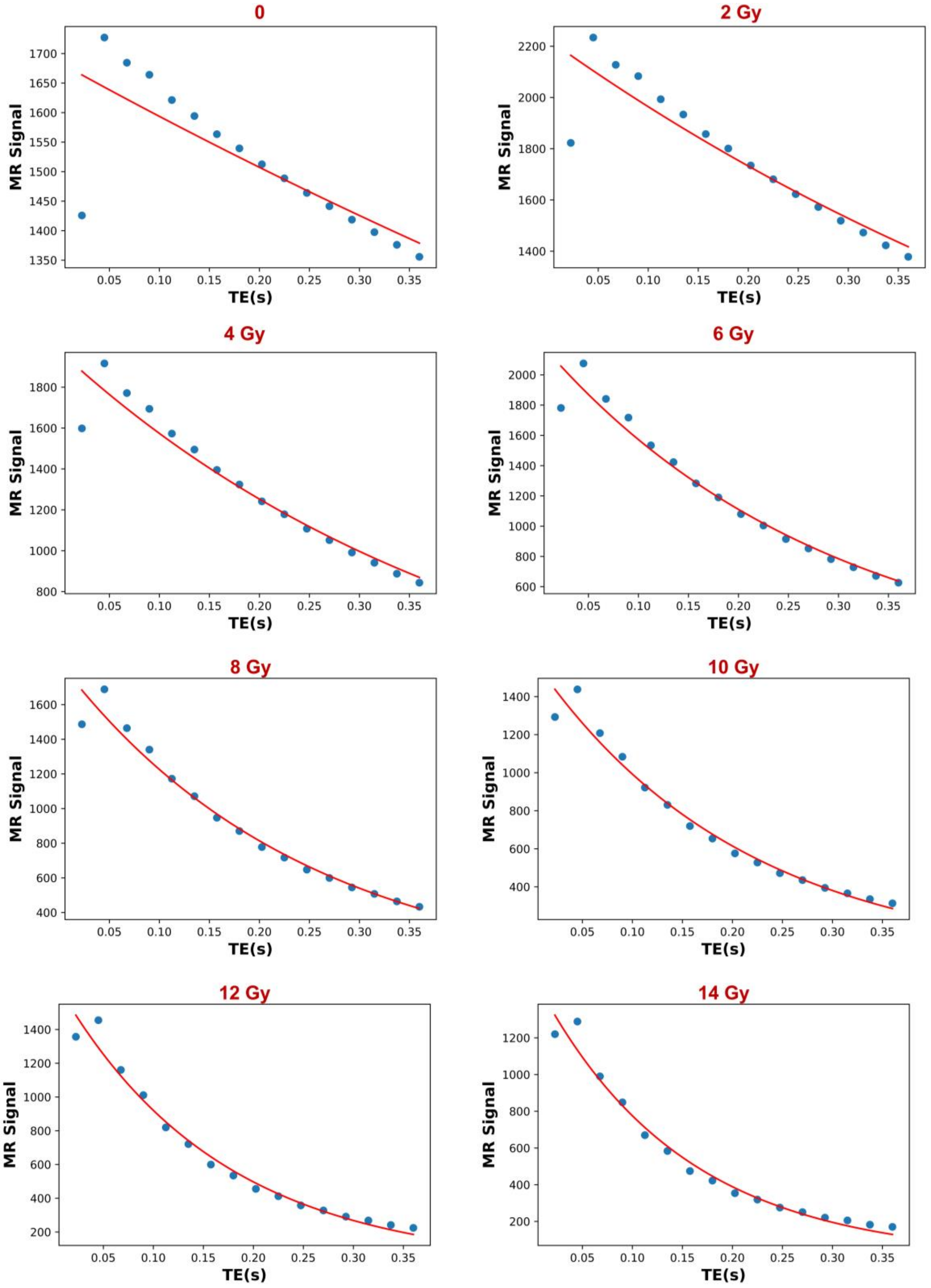

Figure S8. The mono-exponential curves demonstrate the relationship between MR signals and all 16 points of TE(s) for combination irradiation (as an example) for different absorbed doses. The first TE (22.5ms) didn't follow the exponential relationship for EBRT, SIRT and combination irradiations. The effect of the first echo is more evident in the curves for vials with lower doses. This figure is just a visualization of this effect, and the first echo was removed in our analysis.
